# An episomal vector-based CRISPR/Cas9 system for highly efficient gene knockout in human pluripotent stem cells

**DOI:** 10.1038/s41598-017-02456-y

**Published:** 2017-05-24

**Authors:** Yifang Xie, Daqi Wang, Feng Lan, Gang Wei, Ting Ni, Renjie Chai, Dong Liu, Shijun Hu, Mingqing Li, Dajin Li, Hongyan Wang, Yongming Wang

**Affiliations:** 1 Institute of Biomedical Sciences, Shanghai Medical College, Fudan University, Shanghai, 200032 China; 2 The State Key Laboratory of Genetic Engineering and MOE Key Laboratory of Contemporary Anthropology, School of Life Sciences, Fudan University, Shanghai, 200438 China; 3 The Key Lab of Reproduction Regulation of NPFPC in SIPPR, Institute of Reproduction & Development in Obstetrics & Gynecology Hospital, Fudan University, Shanghai, 200011 China; 4 Beijing Anzhen Hospital, Beijing Insitute of Heart Lung and Blood Vessel Disease, Capital Medical University, Beijing, 100029 China; 5 Co-innovation Center of Neuro regeneration, Key Laboratory for Developmental Genes and Human Disease, Ministry of Education, Institute of Life Sciences, Southeast University, Nanjing, 210096 China; 6 Co-innovation Center of Neuroregeneration, Jiangsu Key Laboratory of Neuroregeneration, Nantong University, Nantong, 226001 China; 7 Institute for Cardiovascular Science & Department of Cardiovascular Surgery of the First Affiliated Hospital, Soochow University, Soochow, 215007 China; 8 Children’s Hospital of Fudan University, 399 Wanyuan Road, Shanghai, 201102 China

## Abstract

Human pluripotent stem cells (hPSCs) represent a unique opportunity for understanding the molecular mechanisms underlying complex traits and diseases. CRISPR/Cas9 is a powerful tool to introduce genetic mutations into the hPSCs for loss-of-function studies. Here, we developed an episomal vector-based CRISPR/Cas9 system, which we called epiCRISPR, for highly efficient gene knockout in hPSCs. The epiCRISPR system enables generation of up to 100% Insertion/Deletion (indel) rates. In addition, the epiCRISPR system enables efficient double-gene knockout and genomic deletion. To minimize off-target cleavage, we combined the episomal vector technology with double-nicking strategy and recent developed high fidelity Cas9. Thus the epiCRISPR system offers a highly efficient platform for genetic analysis in hPSCs.

## Introduction

Human pluripotent stem cells (hPSCs), including human embryonic stem cells (hESCs)^1^ and the closely related human induced pluripotent stem cells (iPSCs)^2^, hold great promise for modeling human organ development, analyzing disease mechanisms, and developing potential therapies. However, to fulfill these potential applications, it is crucial to develop methods for efficient and controllable genetic manipulation.

The advent of genome editing technology allows the direct introduction of specific genetic mutations into the hPSCs^3, 4^. Clustered regularly interspaced short palindromic repeats/CRISPR-associated nuclease 9 (CRISPR/Cas9), adapted from microbial adaptive immune defense system^5, 6^, is a recently reported genomic editing tool that is rapidly gaining popularity due to the ease of assembly and high efficiency^3, 4^. In this system, a 20 nt guide RNA (gRNA) directs the Cas9 nuclease to generate site-specific double-stranded breaks (DSBs). The DSBs are typically repaired by the cell’s endogenous DNA repair machinery through non-homologous end-joining (NHEJ), resulting in nonspecific small insertions and deletions (indels) useful for generating loss-of-function mutations^3, 4^. The DSBs can also be repaired by homology-directed repair (HDR) using an introduced DNA repair template, such as a double-stranded DNA donor plasmid or a single-stranded oligo DNA nucleotide (ssODN), leading to knock-in of precise mutations or reporters^7–9^. The genome editing through HDR pathway is less efficient than NHEJ and often requires drug selection of the successful knock-in cells^7, 8^.

Although high efficiency of gene knockout has been achieved in many immortalized tumor cell lines^10^, it has remained a challenge in hPSCs which are more difficult to transfect and less resilient to DNA damage^11^. The genome editing is generally performed by transient expression of Cas9 and a gRNA^3, 11, 12^. The typical efficiencies of gene knockout have been reported to be 1–25% without any subsequent selection steps in hPSCs^3, 11, 13–16^. It is laborious and time-consuming to isolate the homozygous knockout hPSC clones with current efficiency. For example, Gonzalez *et al*. failed to isolate homozygous *GATA6* knockout mutants out of 384 clones^9^. Several strategies have been developed to improve the editing efficiency. Transfection followed by fluorescence-assisted cell sorting for Cas9_GFP+ cells or drug selection could significantly increase editing efficiency with 10–88% indel rates^17–21^. Generation of SpCas9-expressing cell lines is another strategy to increase genome editing efficiency (24–91%)^9, 22^.

Recently, Li *et al*. have achieved high efficiency (8–76%) of genome editing by using an episomal vector to express Cas9 and gRNA^23^. In this study, we used episomal CRISPR/Cas9 system for efficient genome editing in hPSCs. The vector contains OriP/EBNA1 components originated from Epstein-Bar virus, which can drive plasmid duplication once per cell division in eukaryotes^24^, allowing Cas9 and gRNAs persistently expressed in cells; the vector contains a puromycin resistance gene, allowing enrichment of transfected cells by drug selection. The episomal vector is lost with a rate of 3–6% per cell generation after termination of the drug selection^25^, allowing removal of exogenous genes. The epiCRISPR system enables up to 100% gene knockout, which could facilitate efficient genetic analysis in hPSCs.

## Results

### Establishment of an episomal vector-based CRISPR/Cas9 system

**Figure 1:**
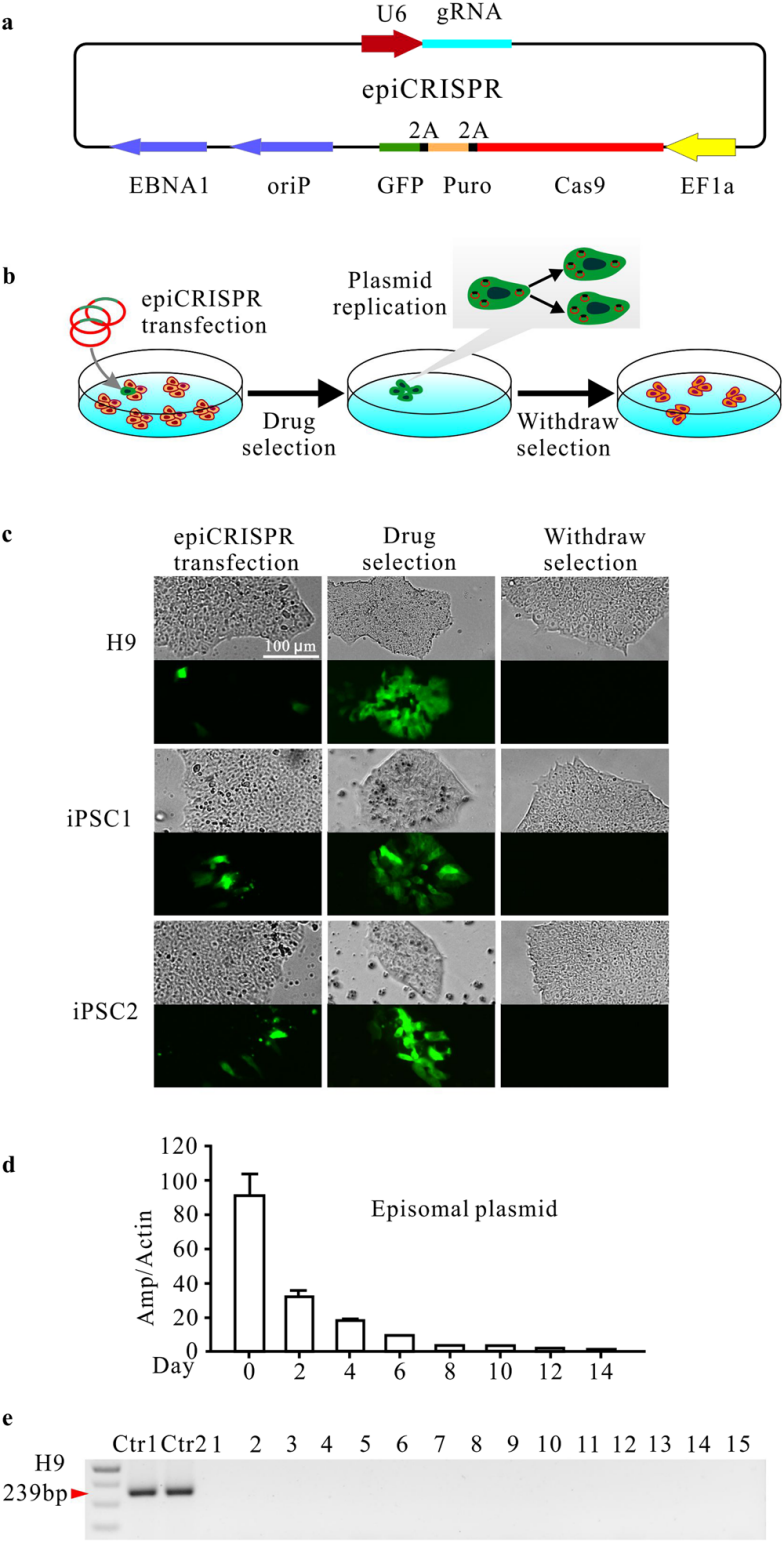
An epiCRISPR system for exogenous gene-free genome editing in hPSCs. (**a**) Schematic of the epiCRISPR system design. The vector contains a U6 promoter-driven gRNA scaffold, an EF1a promoter-driven Cas9 fused to puromycin resistance gene and GFP with P2A peptides, and OriP/EBNA1 elements for the plasmid replication in eukaryocytes. Puro, Puromycin resistance gene. (**b**) Schematic of genome editing with epiCRISPR system. The epiCRISPR vector is transfected into hPSCs followed by drug selection. Only the transfected cells can survive and proliferate. The epiCRISPR vector can replicate in hPSCs and can be partitioned to *daughter cells*. In the absence of drug selection, the epiCRISPR vector can be lost, allowing edited cells free of exogenous gene expression. (**c**) The epiCRISPR system for stable gene expression in hPSCs. The left panel shows the epiCRISPR vector delivered into hPSCs with lipid-mediated transfection. The middle panel shows the epiCRISPR vector can replicate during hPSC proliferation under drug selection. The right panel shows the single cell-derived clones lost epiCRISPR vector without drug selection. (**d**) Episomal vector was decreased within cells over time after withdrawing puromycin selection. The plasmid was tested every two days by quantitative PCR (n = 3, error bars show mean ± S.D.). (**e**) The absence of episomal vector in single cell-derived hESC clones was confirmed by PCR targeting ampicillin sequence. Ctr1 is the positive control amplified from the epiCRISPR plasmid DNA; Ctr2 is the positive control amplified from the pooled hESCs containing epiCRISPR vector.

The current genome editing technology relies on transient expression of Cas9 and a specific gRNA. We expected that the extension of the Cas9 and gRNA expression would increase the modification efficiency. We designed an all-in-one OriP/EBNA1-based vector, which we named epiCRISPR, expressing a gRNA, Cas9, puromycin resistance gene (for enrichment of the transfected cells through drug selection) and GFP (for tracking transfection efficiency) separated by self-cleaving T2A peptide (Fig. 1a and b).

First, we analyzed the capacity of the epiCRISPR system for supporting gene expression in hPSCs (all the experiments in this study were performed in one hESC line (H9) and two iPSC lines). The plasmid was delivered into the cells using lipid-based transfection and low transfection efficiency was observed based on GFP expression (Fig. 1c, left panel). The puromycin selection was started 24 hours post-transfection. While the GFP-negative cells kept dying, the GFP-positive cells kept robust proliferating (Fig. 1c, middle panel). The selection is generally complete in 5–7 days. The consistent supply of puromycin is required to prevent epiCRISPR vector loss during cell proliferation.

The ideal modified cells should be free of genomic integration and exogenous gene expression. To analyze if the epiCRISPR vector can be removed, we stopped drug selection and measured the amount of episomal vector every two days by quantitative polymerase chain reaction (qPCR). The episomal vector was decreased dramatically in the first week (Fig. 1d). We disassociated cells into single cells for colony formation for 15 days. All the single cell-derived clones are GFP-negative (Fig. 1c, right panel), and the vector is undetectable by PCR (Fig. 1e), indicating that the episomal vector can be efficiently removed. In summary, the epiCRISPR system allows modified cells free of genomic integration and exogenous gene expression in hPSCs.

### The epiCRISPR system significantly promotes gene knockout in hPSCs

**Figure 2:**
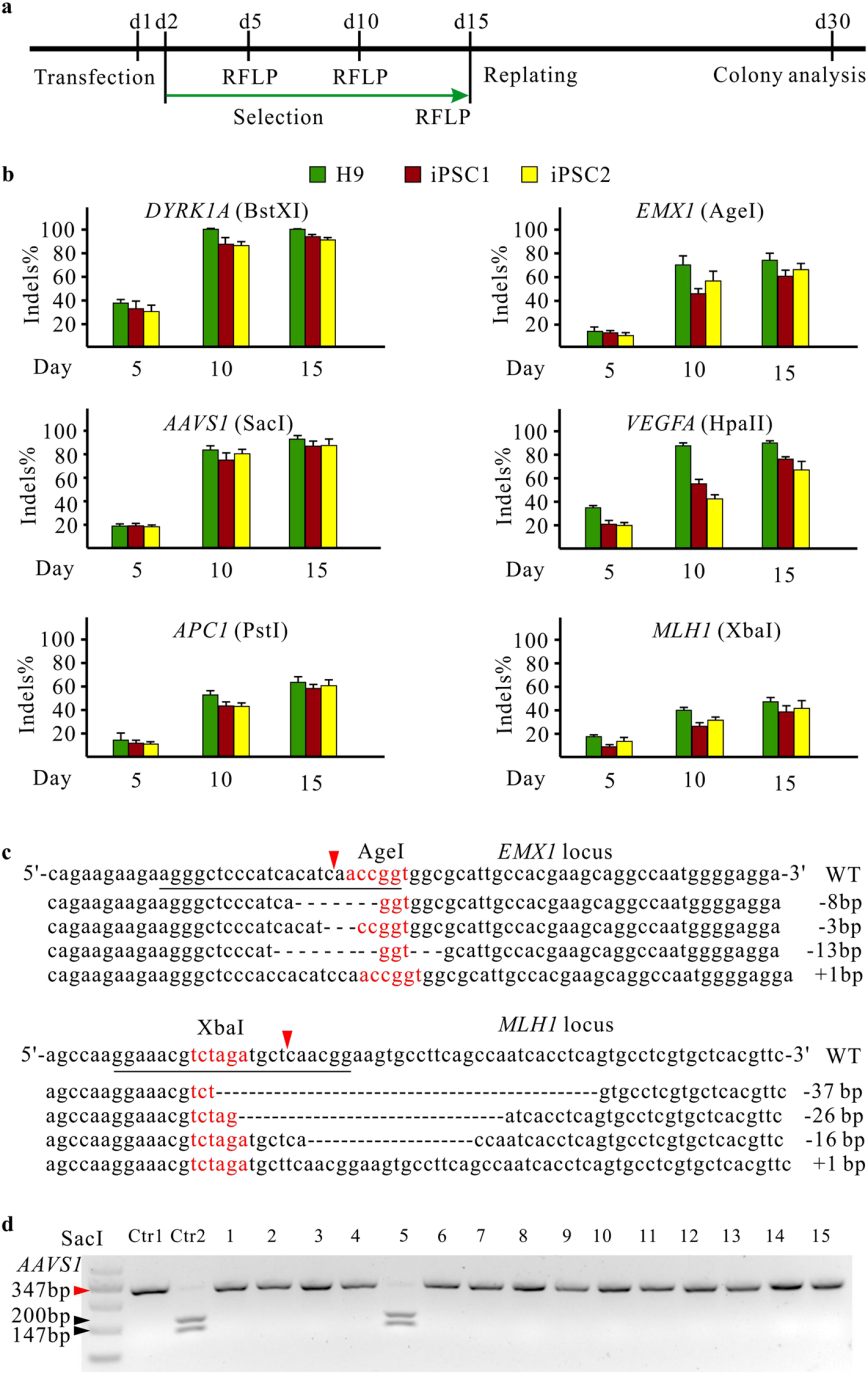
The epiCRISPR system for efficient gene knockout. (**a**) Schematic representation of the experimental procedure. (**b**) RFLP analysis of the indel rates generated by the epiCRISPR system at *DYRK1A*, *EMX1*, *AAVS1*, *VEGFA*, *APC1* and *MLH1* loci in hPSCs (n = 3, error bars show mean ± S.D.). (**c**) Representative indel sequences at *EMX1* and *MLH1* sites in hESCs. The AgeI and XbaI restriction sequences are shown in red. The red triangles indicate Cas9 cutting site. (**d**) RFLP analysis of single hESC-derived clones modified at *AAVS1* locus. Ctr1 is the PCR band from unmodified cells without digestion. Ctr2 is the PCR band from unmodified cells with SacI digestion. The red triangle indicates the epiCRISPR-modified PCR bands; the black triangle indicates unmodified PCR bands.

Next, we analyzed the capacity of the epiCRISPR system for gene knockout in hPSCs. We designed a panel of gRNAs targeting six loci (*DYRK1A*, *EMX1*, *AAVS1*, *VEGFA*, *APC1* and *MLH1*) located on different chromosomes. These targeted loci contain restriction sites so that we can assess the modification efficiency with Restriction Fragment Length Polymorphism (RFLP) assay. We observed an increasing insertion and deletion (indel) rates over time (Supplementary Figures S1 and S2). For example, the indel rates for *AAVS1* locus were 19% at day 5, 83% at day 10 and 93% at day 15 after transfection in hESCs (Fig. 2a and b; Supplementary Table S1). We observed that the indel rates varied amount three cell lines for the VEGFA locus at day 10. The possible reason is that these cell lines have different origin and VEGFA locus might be associated with different epigenetic modification which influenced Cas9 accessibility. The sequencing results revealed even higher indel rates (27/30 = 90% for *EMX1* locus; 14/17 = 82% for *MLH1* locus) because a portion of the restriction sequence was not influenced by indels (Fig. 2c). The indel rates varied from 82 to 100% at day 15 depending on gRNAs and cell lines. We analyzed 15 single cell-derived clones modified at *AAVS1* locus and 14 clones were biallelic knockout (Fig. 2d). We further investigated if the episomal vector has advantage over transient plasmid with the same editing time. We performed side-by-side comparison of the epiCRISPR system to the popularly used editing plasmids for three gRNAs with either Puromycin selection (pX459 plasmid) or cell sorting (pX458 plasmid)^26^. The epiCRISPR system and the transient plasmids generated comparable efficiency of editing at day 2 and day4 (Supplementary Figure S3), demonstrating that the episomal vector could not increase genome editing without elongating editing time.

**Figure 3:**
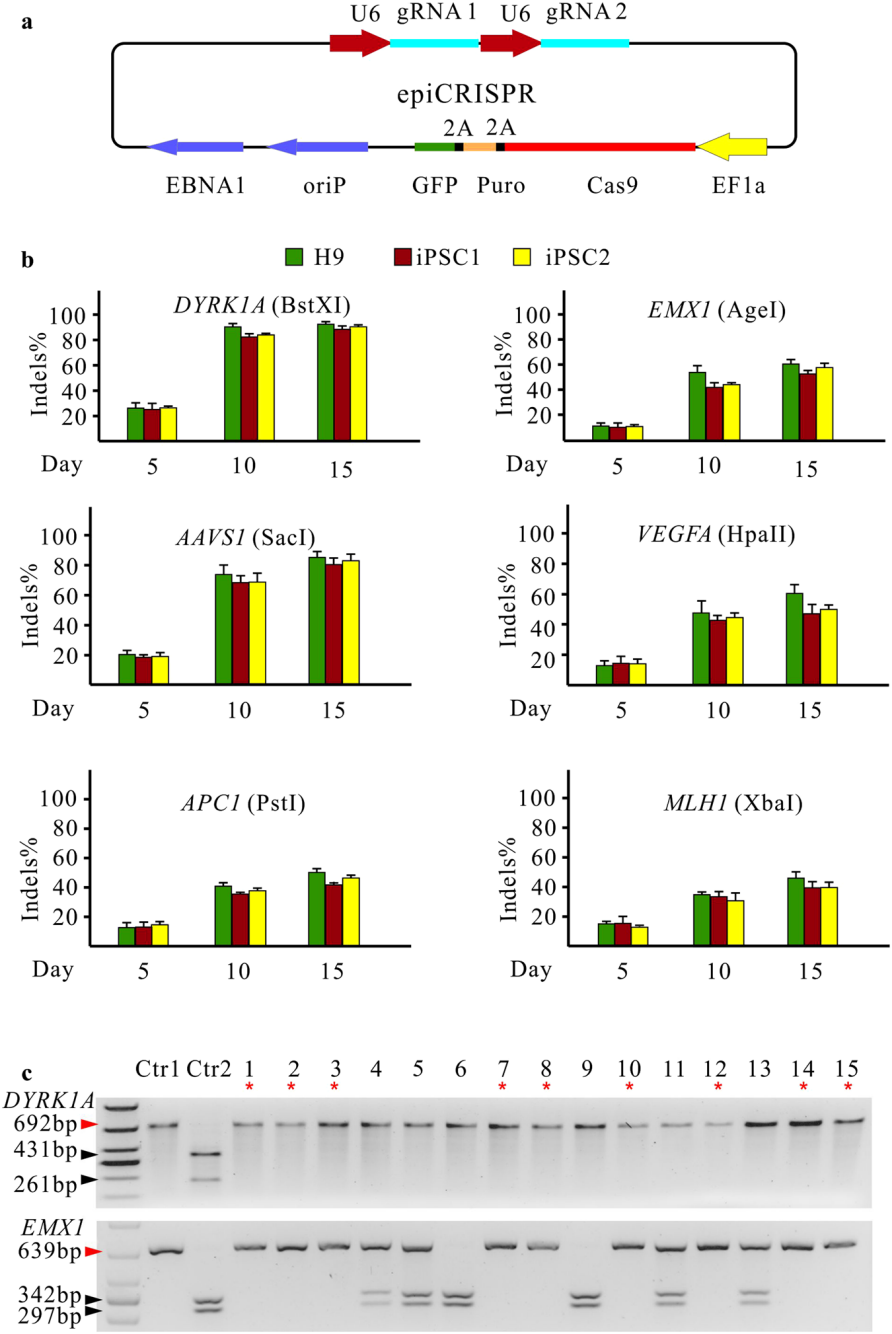
The epiCRISPR system for efficient double-gene knockout. (**a**) Expression of two gRNAs on the epiCRISPR vector for double-gene knockout. (**b**) RFLP analysis of the indel rates generated by the epiCRISPR system with gRNA multiplexed targeting *DYRK1A* & *EMX1*, *AAVS1* & *VEGFA* and *APC1* & *MLH1* in hPSCs (n = 3, error bars show mean ± S.D.). (**c**) RFLP analysis of single cell-derived clones multiplexed targeting *DYRK1A* and *EMX1* loci. Ctr1 is the PCR band from unmodified cells without digestion. Ctr2 is the PCR band from unmodified cells with digestion (BstXI for *DYRK1A* and AgeI for *EMX1*). Red triangles indicate the epiCRISPR-modified PCR bands; black triangles indicate unmodified PCR bands; the red asterisks indicate the homozygous knockout for both genes.

To determine the capacity of the epiCRISPR system for double-gene knockout in hPSCs, we expressed two gRNAs on the epiCRISPR vector (Fig. 3a). Multiplexed targeting of *DYRK1A* & *EMX1*, *AAVS1* & *VEGFA* and *APC1* & *MLH1* achieved similar indel rates to the single-gene knockout at day 15 (Fig. 3b; Supplementary Figure S4 and Table S1). We analyzed 15 single cell-derived clones modified at *DYRK1A* & *EMX1* loci in the hESCs and 9 clones were homozygous knockout for both genes (Fig. 3c). Interestingly, all 15 clones were homozygous knockout at the *DYRK1A* locus. Therefore, the epiCRISPR system is a powerful platform for single- and double-gene knockout in hPSC lines.

### The epiCRISPR system for efficient genomic deletions in hPSCs

**Figure 4:**
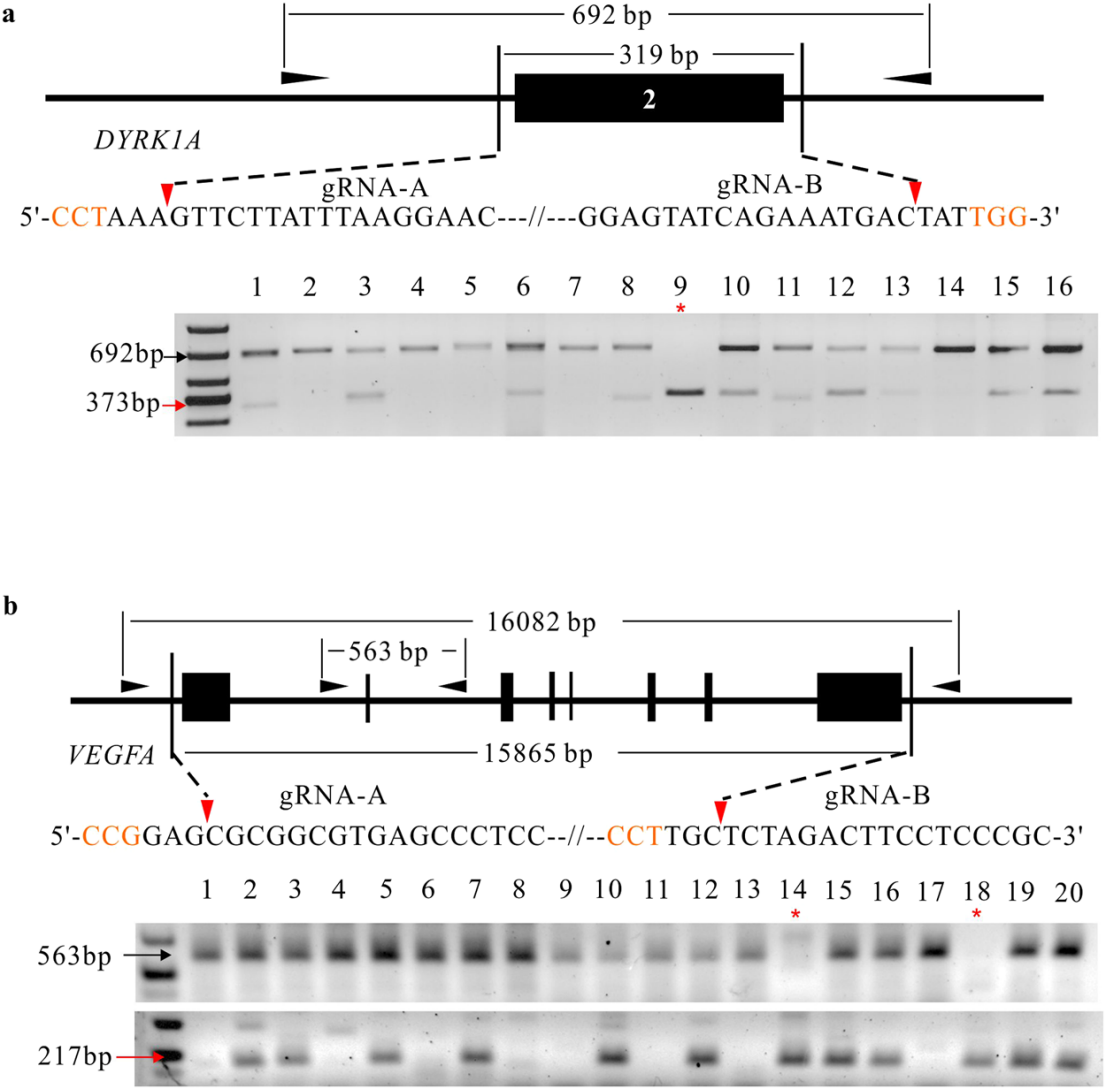
The epiCRISPR system for efficient genomic deletions. (**a**) The strategy for deletion of the 2^nd^ exon of *DYRK1A*. A 373-bp PCR band will be present when deletion occurs (indicate by red arrow). A representative gel picture was shown. (**b**) The strategy for deletion of the full-coding sequence of *VEGFA*. Two pairs of primers were designed. One pair is to detect the non-deletion allele (563-bp) and one pair is to detect deletion events (217-bp, indicate by red arrow). The black triangles indicate the primers for the genotyping PCR reaction. The outer primers work only when deletion occurs. The PAM sequence is shown in orange; the red triangles indicate Cas9 cutting site; red arrows indicate the epiCRISPR-modified PCR bands; black arrows indicate unmodified PCR bands; the red asterisks indicate the homozygous knockout for both genes.

To determine the capacity of the epiCRISPR system for genomic deletions in hPSCs, we designed paired gRNAs targeting the 2^nd^ exon of *DYRK1A* for 319-bp deletion in hESCs. Of 16 clones analyzed, nine clones were monoallelic deletion and one clone was biallelic deletion (Fig. 4a). To determine if the epiCRISPR system can delete larger genomic DNA fragment, we designed paired gRNAs to delete the full coding sequence of *VEGFA* (16082 bp) in hESCs. Of 20 clones analyzed, 10 clones were monoallelic deletion and two clones were biallelic deletion (Fig. 4b). Therefore, the epiCRISPR system enables efficient generation of homozygous large genomic deletions in hPSCs.

### Off-target analysis of the epiCRISPR system in hPSCs

**Figure 5:**
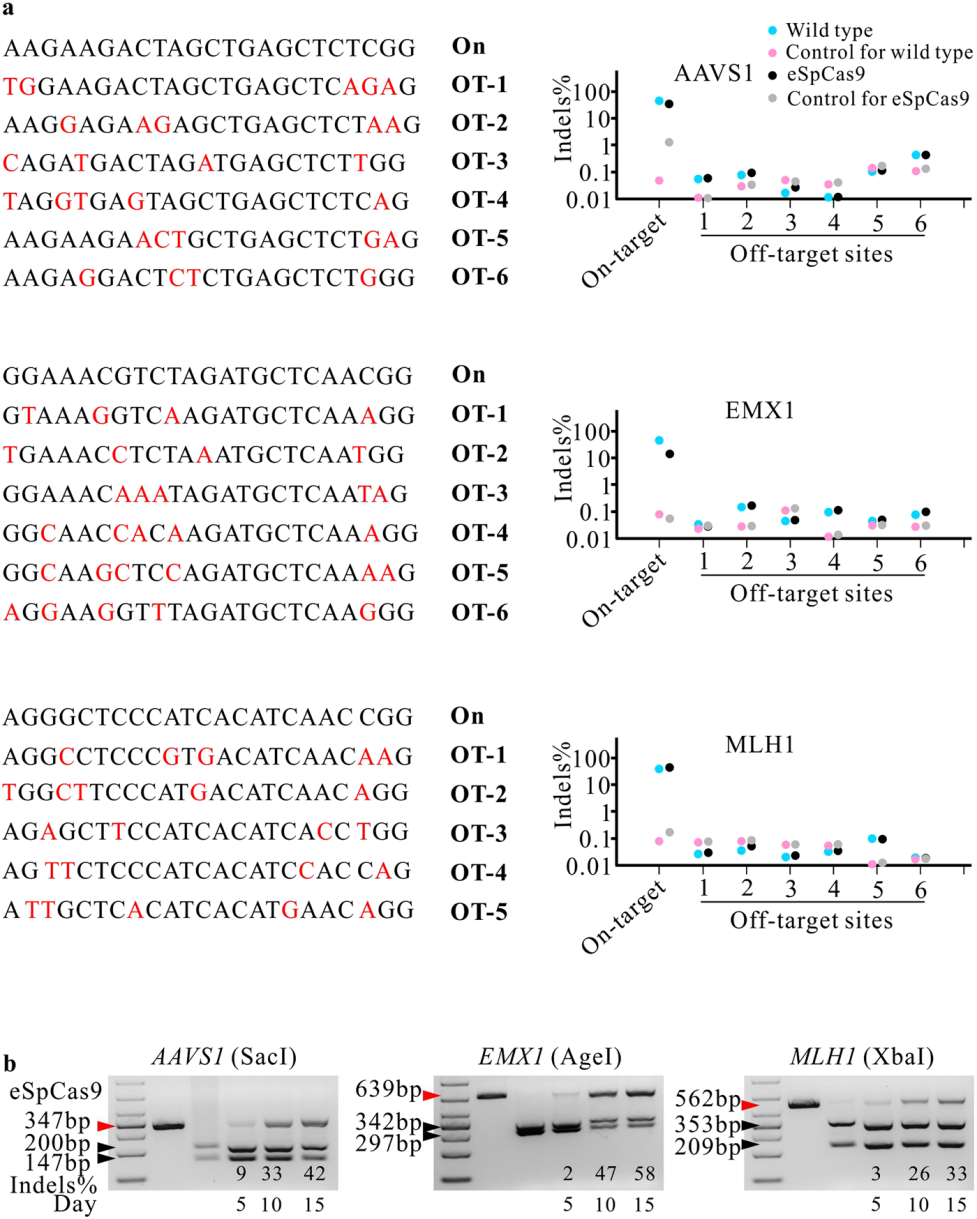
Analysis of off-target cleavage by targeted deep-sequencing. (**a**) The potential off-target sequences are shown on the left; the indel rates are shown on the right. On, on target sequence; OT, off-target sequence. The unmatched nucleotides are shown in red. (**b**) RFLP analysis of eSpCas9 cleavage at *AAVS1*, *EMX1* and *MLH1* sites. Red triangles indicate the epiCRISPR-modified PCR bands; black triangles indicate the unmodified PCR bands.

In previous reports, off-target mutations could be induced during genome editing^27, 28^. The epiCRISPR system requires long-term editing which may increase off-target effects. We analyzed a panel of potential off-target sites by using targeted deep sequencing for the locus of the *AAVS1*, *MLH1* and *EMX1* sites. These potential off-target sites were identified using the online tool (http://crispr.mit.edu/) that developed by Feng Zhang lab. Although we have edited cells for 15 days, the off-target indel rates (0.075 ± 0.096%) were at the background level (0.049 ± 0.038%; P = 0.298, Fig. 5a). Recently, Slaymaker *et al*. developed a new version of SpCas9 (eSpCas9) with enhanced specificity^29^. We replaced wild-type SpCas9 with eSpCas9 and got epiCRISPRe vector. The eSpCas9 showed lower on-target cleavage activity compared to the wild-type SpCas9 as previously reported (Fig. 5b)^29^. Similar to the SpCas9, the off-target indel rates (0.084 ± 0.096%) of eSpCas9 were at the background level (0.057 ± 0.047%; P = 0.285, Fig. 5a). Notably, OT-6 locus for the *AAVS1* showed higher indel rates for both SpCas9 (0.43%) and eSpCas9 (0.43%). These indels are probably due to sequencing/PCR error because eSpCas9 could not tolerate three mismatches^29^. Our study did not show the advantage of the eSpCas9 because the on-target sequences are very specific in the genome. The eSpCas9 could be useful for the locus that has high sequence similarity in the genome.

**Figure 6:**
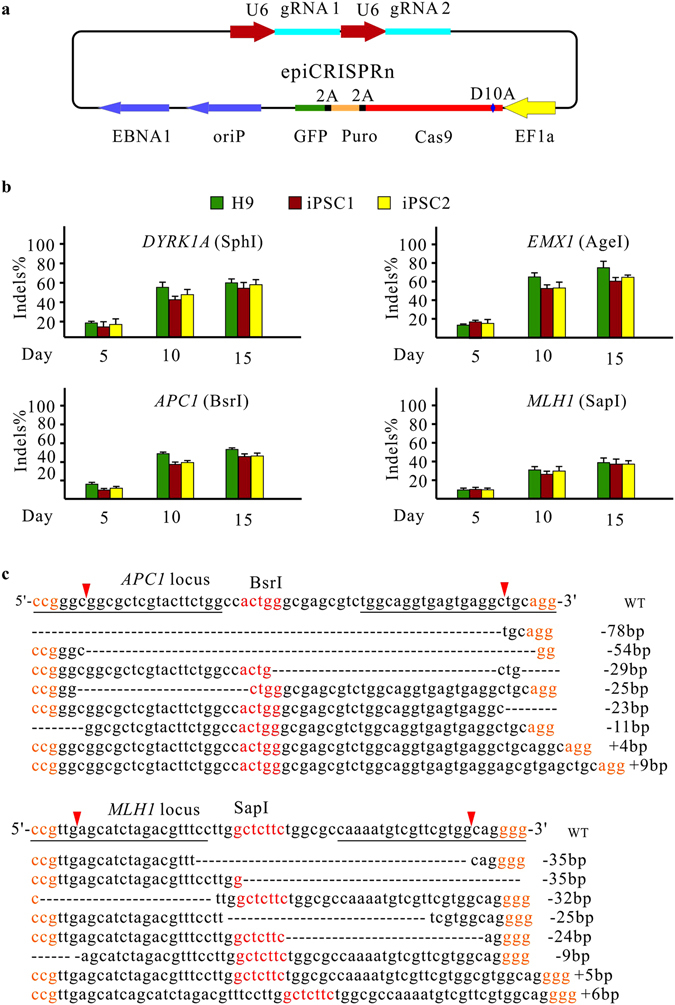
An epiCRISPRn system for efficient double-nicking. (**a**) Schematic of the epiCRISPRn vector design. A D10A mutation was introduced into the epiCRIPSR vector. (**b**) RFLP analysis of the indel rates generated by the epiCRISPRn system targeting *DYRK1A*, *EMX1*, *AAVS1*, *VEGFA*, *APC1* and *MLH1* loci (n = 3, error bars show mean ± S.D.). (**c**) Representative indel sequences generated by the epiCRISPRn at *APC1* and *MLH1* loci. The size of indels is shown on the right. The gRNA sequence is underlined; the PAM sequence is shown in orange; the restriction sites are shown in red, the red triangles indicate Cas9 cutting site.

In addition to high-fidelity Cas9, we combined the episomal vector with double-nicking strategy. We named this system epiCRISPRn. The double-nicking strategy was originally developed by Ran *et al*.^27^. In this strategy, the D10A mutant Cas9 nickase (Cas9n) is specified by a pair of gRNAs to simultaneously introduce single-stranded nicks on both strands of the target DNA, significantly increasing the specificity of genome editing^27^. The epiCRISPRn was created by introducing a point mutation (D10A) into the Cas9 on epiCRISPR vector (Fig. 6a). A panel of paired gRNAs was designed to target four loci (*DYRK1A*, *EMX1*, *APC1* and *MLH1*). Fifteen days after transfection, RFLP assay revealed that the indel rates were 33–80% (Fig. 6b; Supplementary Figure S5 and Table S1). The sequencing results revealed much higher indel rates (17/20 = 85% for *APC1* locus; 16/20 = 80% for *MLH1* locus), because the indels shifted between two cutting sites and could not efficiently disrupt the restriction site (Fig. 6c). The majority of the modifications are small deletions (average 29.8 bp, range: 6–78 bp). In summary, the epiCRISPRn system enables efficient genome editing with double-nicking strategy in hPSCs.

### The epiCRISPR system does not influence pluripotency and karyotype of hPSCs

Further analysis revealed that the modified hESCs displayed normal morphology and karyotype (Supplementary Figure S6A and B). Modified hESCs maintained their pluripotent state as indicated by the expression of pluripotency markers (Supplementary Figure S7A). In addition, these cells were capable of differentiation into all three germ layers both *in vivo* and *in vitro* (Supplementary Figure S7B and C).

## Discussion

In the present study, we have developed an epiCRISPR system for highly efficient genome editing in hPSCs. Through extension of Cas9 and gRNA expression using an episomal vector, we have achieved up to 100% indel rates. We have further shown that the epiCRISPR system supports efficient double-gene knockout and large genomic deletions. To minimize the off-target cleavage, we have also combined episomal vector technology with high-fidelity Cas9 and double-nicking strategy. Importantly, the episomal vector can be removed after genome editing, allowing edited cells free of exogenous gene expression. Our results demonstrate that the epiCRISPR system is a powerful tool for the gene knockout in the hPSCs.

Loss-of-function studies in hPSCs require efficient genome editing to disrupt genes of interest. Several groups have shown that 10–88% indel rates could be achieved by enriching transfected hPSCs with cell sorting or drug selection^17–21^. In this study, we have developed an alternative strategy for efficient genome editing. The epiCRISPR system promotes genome editing by two ways. First the episomal vector allows continuously selecting transfected cells. Several successfully transfected cells are enough for the efficient genome editing. Since the transfection efficiency is not an obstacle for the editing, the epiCRISPR system allows lipid-mediated transfection which is convenient and less cytotoxic for the hPSCs^9^. Second, the episomal vector allows long-term genome editing. Our results show that the editing efficiency increases over time. In theory, the majority of gRNAs can generate indel rates up to ~100% if the editing time is long enough. In this study we have achieved 82–100% indel rates with 15 days’ editing. Notably, not all of the indel mutations could fully disrupt gene functions. The epiCRISPR system also enables to generate double-gene knockout cells. Although we did not try in this study, the epiCRISPR system is likely to support triple- or more gene knockout in one-step. This powerful tool would facilitate to investigate gene-gene interactions in the hPSCs.

In addition to indels, the epiCRISPR system also supports large genomic deletions in hPSCs. Deletions have potential advantages as compared to indels given the predictability of loss-of-function and utility for the study of non-coding elements. In this study, we have showed two examples of genomic deletions. The biallelic deletions could be isolated by analysis of 20 clones. The genomic deletion efficiency depends on individual gRNA activity. We did not optimize the gRNA activity in this study. Recent genome-wide studies have shown that majority of the human genome is comprised of potential functional noncoding elements, such as intergenic or intronic cis-regulatory modules, miRNA clusters, or lincRNAs^30, 31^. The epiCRISPR system facilitates to study noncoding elements in hPSCs.

Several investigations have shown that CRISPR/Cas9 can generate off-target cleavage in somatic cells^10, 27–29^. These investigations have largely focused on sites with high sequence similarity to the on-target site and have documented indel rates as high as 50% at individual off-target sites. In contrast, two groups have investigated off-target effects at sites with low sequence similarity to the on-target site in hPSCs^32, 33^. They analyzed several CRISPR/Cas9-edited single cell-derived hPSC clones by whole-genome sequencing, but they did not identify any off-target cleavage, demonstrating the specificity of the CRISPR/Cas9 in the hPSCs. The on-target sites we chose in this study have no off-target sites with high sequence similarity. Consistent with previous studies, we did not observe off-target cleavage at 18 sites for both wild-type and high-fidelity Cas9, although we have expressed Cas9 and gRNAs for 15 days. CRISPR/Cas9 may generate indels at off-target site with high sequence similarity to the on-target site. In this case, the high-fidelity Cas9 or double-nicking strategy could be used to reduce off-target cleavage. When we were preparing the manuscript, another group reported that the episomal CRISPR/Cas9 system could work efficiently in HeLa cells and mouse iPSCs^23^. Taken together, the epiCRISPR system offers a powerful platform for highly efficient genome editing in a variety of cells.

## Materials and Methods

### Cell culture and maintenance of human pluripotent stem cells

Human ESCs (WA09, Wicell, Madison, WI) and iPSCs were cultured on Matrigel-coated plates (ESC qualified, BD Biosciences, San Diego, CA) using hESC mTeSR-1 cell culture medium (StemCell Technologies, Vancouver, Canada) under conditions of 37 °C, 95% air, and 5% CO_2_ in a humidified incubator, as previously described^8^. Results for subsequent experiments are based on 1 hESC line (WA09) and 2 iPSC lines.

### Plasmids and oligonucleotides

The epiCRISPR plasmid: the SpCas9, puromycin resistance gene and copGFP were co-expressed from an EF1a promoter as a single protein separated by self-cleaving P2A peptides; the OriP/EBNA1 components driving plasmid duplication were derived from pREP9 vector (Invitrogen); gRNA was expressed from a human U6 promoter. The epiCRISPRe plasmid is the same as epiCRISPR except that three mutations (K848A/K1003A/R1060A) were introduced into the SpCas9, and the human U6 promoter was replaced by mouse U6 promoter so that it can express gRNA starting with both G and A. To express a gRNA, the oligonucleotide duplexes were cloned into BspQI restriction sites of the epiCRISPR plasmid. The epiCRISPRn plasmid is the same as epiCRISPR except that a D10A mutation was introduced into the SpCas9. A detailed protocol for cloning two gRNAs into vector was provided in Supplementary Figure S8. The amino acid substitutions were generated by standard PCR. DNA sequence of plasmids used in this study can be found in the Supplementary Figure S9. gRNA target sites are available in Supplementary Table 2, and oligonucleotides used in this study can be found in Supplementary Table 3.

### Genome editing with epiCRISPR system

4 × 10^5^ human ESCs or iPSCs were disassociated and cultured in a 6-well plate (Sigma Aldrich, St. Louis, MO) with mTeSR-1 cell culture medium for 24 hours. At day 1, two μg of the epiCRISPR plasmid were transfected into cells by using lipofectamine 3000 (Life Technologies) based on online protocol. At day 2, cells were selected by puromycin (0.2–0.5 μg/ml). At day 5, day 10 and day 15, genomic DNA was extracted from cells using QuickExtract (Epicentre) following the manufacturer’s protocol. The gRNA targeting sites were PCR-amplified using Q5 High-Fidelity DNA polymerase (NEB). The PCR products were purified using QiaQuick Spin Column (QIAGEN) following the manufacturer’s protocol. The purified PCR products were digested with restriction enzymes (NEB) for 4 hours and then run them on agarose gel. For analysis of single cell-derived clones, the cells were disassociated into single cells at day 15 post- transfection, and seeded them onto the Matrigel-coated plates with puromycin-free mTeSR-1 medium for 15 days. Individual colonies were picked and genotyped.

### Off-target cleavage analysis

Fifteen days after genome editing, genomic DNA was extracted using the QuickExtract DNA Extraction Solution (Epicenter) following the manufacturer’s protocol. Potential off-target sites predicted by an online tool (http://crispr.mit.edu/) were amplified using Q5 High-Fidelity DNA polymerase (NEB) with the primers listed in Supplementary Table 3. The genome sequence spanned by the corresponding primer pairs were extracted from human genome sequence (Hg19, GRChr37), acting as the reference sequence for analyzing sequence variation caused by genome editing. PCR products were generated for each on- and off-target site from ~100 ng of genomic DNA extracted from hESCs. PCR products were purified with QIAguick Gel Extraction Kit, normalized in concentration, pooled together, phosphorylated at 5′ end, added dA-Tailing and Y-Shape adapter. The resultant products were PCR-amplified with KAPA HIFI Hotstart Readymix (KAPA Biosystems) and primers (100 nM) carrying Illumina sequence adaptors. PCR products were purified with QIAquick Gel Extraction Kit (QIAGEN), and sequenced via 150-bp paired-end sequencing on an Illumina MiSeq instrument. To avoid the possible errors in the sequencing process, each read was first filtered by removing the nucleotides with a quality score smaller than 30, and the following nucleotides were also removed. The resulted high-quality reads were mapped to the reference human genome sequence using pairwise sequence alignment algorithm, BLAT (http://genome.ucsc.edu)^34^, with default parameters. The number of reads with indels to the reference sequence that were 15-bp adjacent to the predicted cleavage site were counted, and divided by the total aligned reads to get the potential editing percentage. Since most of indels were less than 50-bp, an alternative 30-bp DNA fragment 50-bp away from predicted cleavage site was used as control, and the number of reads with indels was also counted to get the corresponding sequence variation percentage.

### Statistical analysis

All the data are shown as the mean ± S.D. Statistical analyses were conducted using Microsoft Excel. Two-tailed, paired Student’s *t*-tests were used to determine statistical significance when comparing two groups. A value of *p* < 0.05 was considered to be statistically significant.

## Electronic supplementary material

Supplementary Information
