# Author Correction: An episomal vector-based CRISPR/Cas9 system for highly efficient gene knockout in human pluripotent stem cells

**DOI:** 10.1038/s41598-018-36738-w

**Published:** 2018-12-12

**Authors:** Yifang Xie, Daqi Wang, Feng Lan, Gang Wei, Ting Ni, Renjie Chai, Dong Liu, Shijun Hu, Mingqing Li, Dajin Li, Hongyan Wang, Yongming Wang

**Affiliations:** 1 Institute of Biomedical Sciences, Shanghai Medical College, Fudan University, Shanghai, 200032 China; 2 The State Key Laboratory of Genetic Engineering and MOE Key Laboratory of Contemporary Anthropology, School of Life Sciences, Fudan University, Shanghai, 200438 China; 3 The Key Lab of Reproduction Regulation of NPFPC in SIPPR, Institute of Reproduction & Development in Obstetrics & Gynecology Hospital, Fudan University, Shanghai, 200011 China; 4 Beijing Anzhen Hospital, Beijing Insitute of Heart Lung and Blood Vessel Disease, Capital Medical University, Beijing, 100029 China; 5 Co-innovation Center of Neuro regeneration, Key Laboratory for Developmental Genes and Human Disease, Ministry of Education, Institute of Life Sciences, Southeast University, Nanjing, 210096 China; 6 Co-innovation Center of Neuroregeneration, Jiangsu Key Laboratory of Neuroregeneration, Nantong University, Nantong, 226001 China; 7 Institute for Cardiovascular Science & Department of Cardiovascular Surgery of the First Affiliated Hospital, Soochow University, Soochow, 215007 China; 8 Children’s Hospital of Fudan University, 399 Wanyuan Road, Shanghai, 201102 China

Correction to: *Scientific Reports* 10.1038/s41598-017-02456-y, published online 24 May 2017

The authors felt they should cite the following article by Ohashi M, et al. (2015), which is included below as Reference 1.

Specifically, in the Introduction the sentence,

“Recently, Li et al. have achieved high efficiency (8–76%) of genome editing by using an episomal vector to express Cas9 and gRNA^23^.”

should read:

“Recently, two groups have achieved high efficiency of genome editing by using an episomal vector to express Cas9 and gRNA in both human somatic cells and mouse iPSCs^23, 1^.”

In addition, in the Discussion, the sentence:

“When we were preparing the manuscript, another group reported that the episomal CRISPR/Cas9 system could work efficiently in HeLa cells and mouse iPSCs^23^.”

should read:

“Two groups have reported that the episomal CRISPR/Cas9 system could work efficiently in both human somatic cells and mouse iPSCs^23, 1^.”
