# Supplementary material for: An episomal vector-based CRISPR/Cas9 system for highly efficient gene knockout in human pluripotent stem cells
Source: Sci Rep. 2017 May 24;7:2320. doi: 10.1038/s41598-017-02456-y (PMC5443789; doi:10.1038/s41598-017-02456-y)
Supplement: Supplementary file 1 — Supplementary Information [file 41598_2017_2456_MOESM1_ESM.pdf]

An episomal vector-based CRISPR/Cas9 system for highly efficient gene knockout in human pluripotent stem cells

Yifang Xie[1*], Daqi Wang[2*], Feng Lan[4], Gang Wei[2], Ting Ni[2], Renjie Chai[5], Dong Liu[6], Shijun Hu[7], Mingqing Li[3], Dajin Li[3], Hongyan Wang[3,8#], Yongming Wang[2,3#]

SUPPLEMENTARY FIGURES

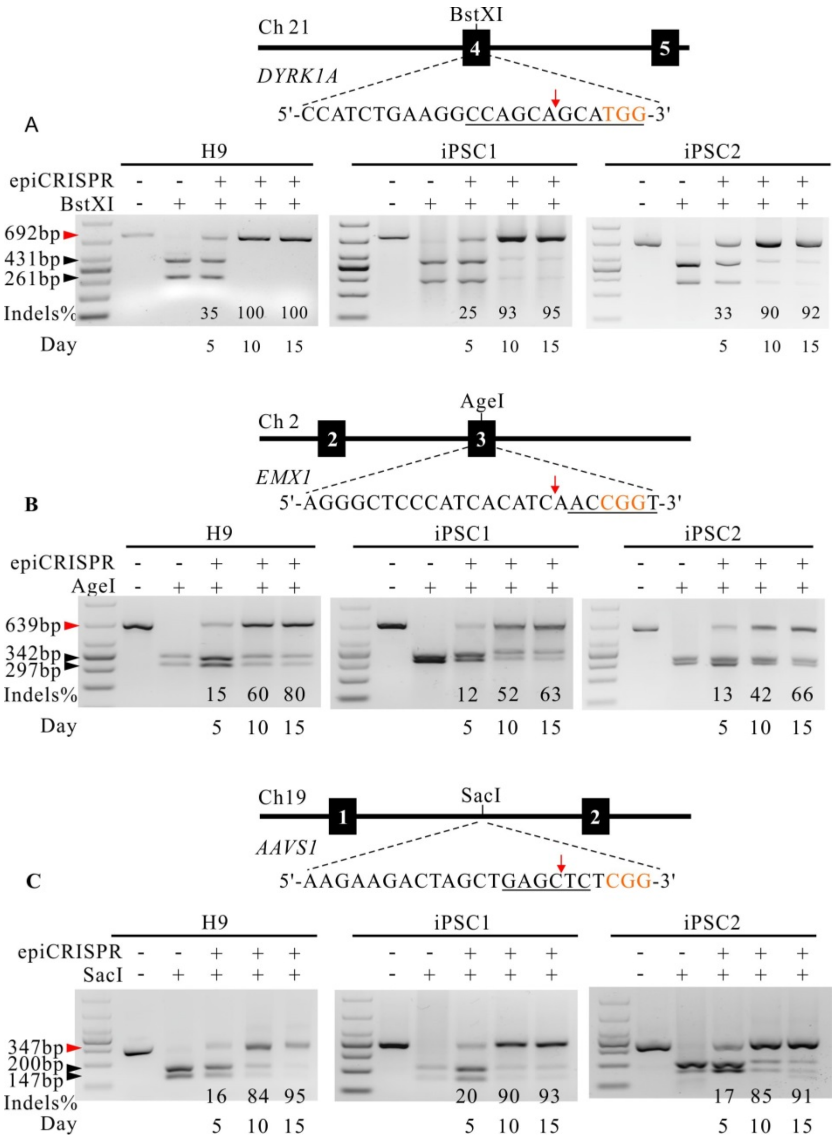

Figure S1. Representative gel analysis of indel rates generated by the epiCRISPR for *DYRK1A*, *EMX1* and *AAVS1* Loci. (A-C) RFLP analysis of indel rates at *DYRK1A* (A), *EMX1* (B) and *AAVS1* (C) loci in hPSCs. The gRNA targeting locus on chromosome is shown by schematic diagrams with the gRNA sequence below. The restriction site is underlined. The PAM sequence is shown in orange; red arrows indicate Cas9 cutting site, red triangles indicate the epiCRISPR-modified PCR bands; black triangles indicate the unmodified PCR bands.

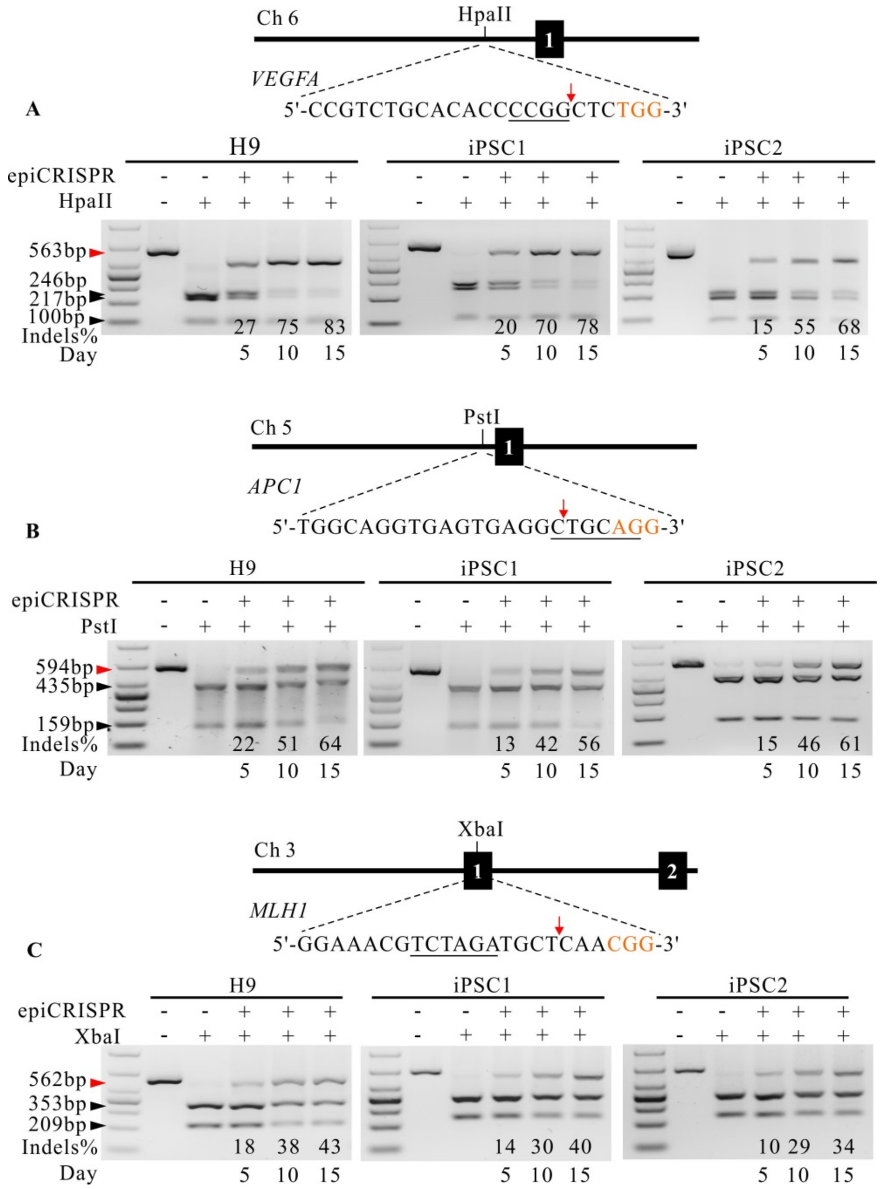

Figure S2. Representative gel analysis of indel rates generated by the epiCRISPR for *VEGFA*, *APC1* and *MLH1* loci. (A-C) RFLP analysis of indel rates at *VEGFA* (A), *APC1* (B) and *MLH1* (C) loci in hPSCs. The gRNA targeting locus on chromosome is shown by schematic diagrams with the gRNA sequence below. The restriction site is underlined. The PAM sequence is shown in orange; red arrows indicate Cas9 cutting site, red triangles indicate the epiCRISPR-modified PCR bands; black triangles indicate the unmodified PCR bands.

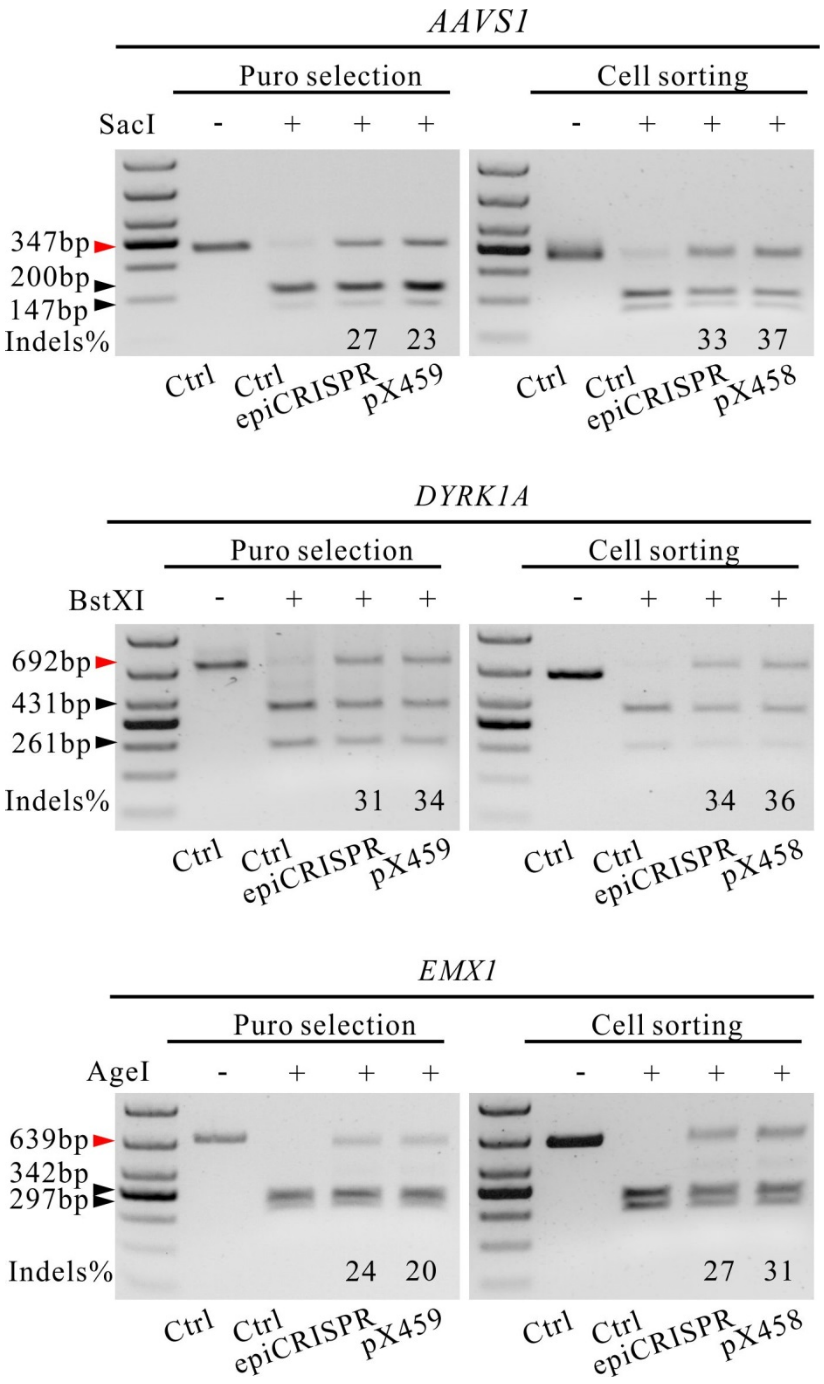

Figure S3. Gel pictures for the comparison of the genome editing efficiency with episomal vector and traditional plasmids in H9 cells. Puromycin selection stared at day 2 after electroporation and lasted for 4 days. Cell sorting was performed 48 h after electroporation. Ctrl is the unmodified DNA. Red triangles indicate the edited PCR bands; black triangles indicate the unedited PCR bands.

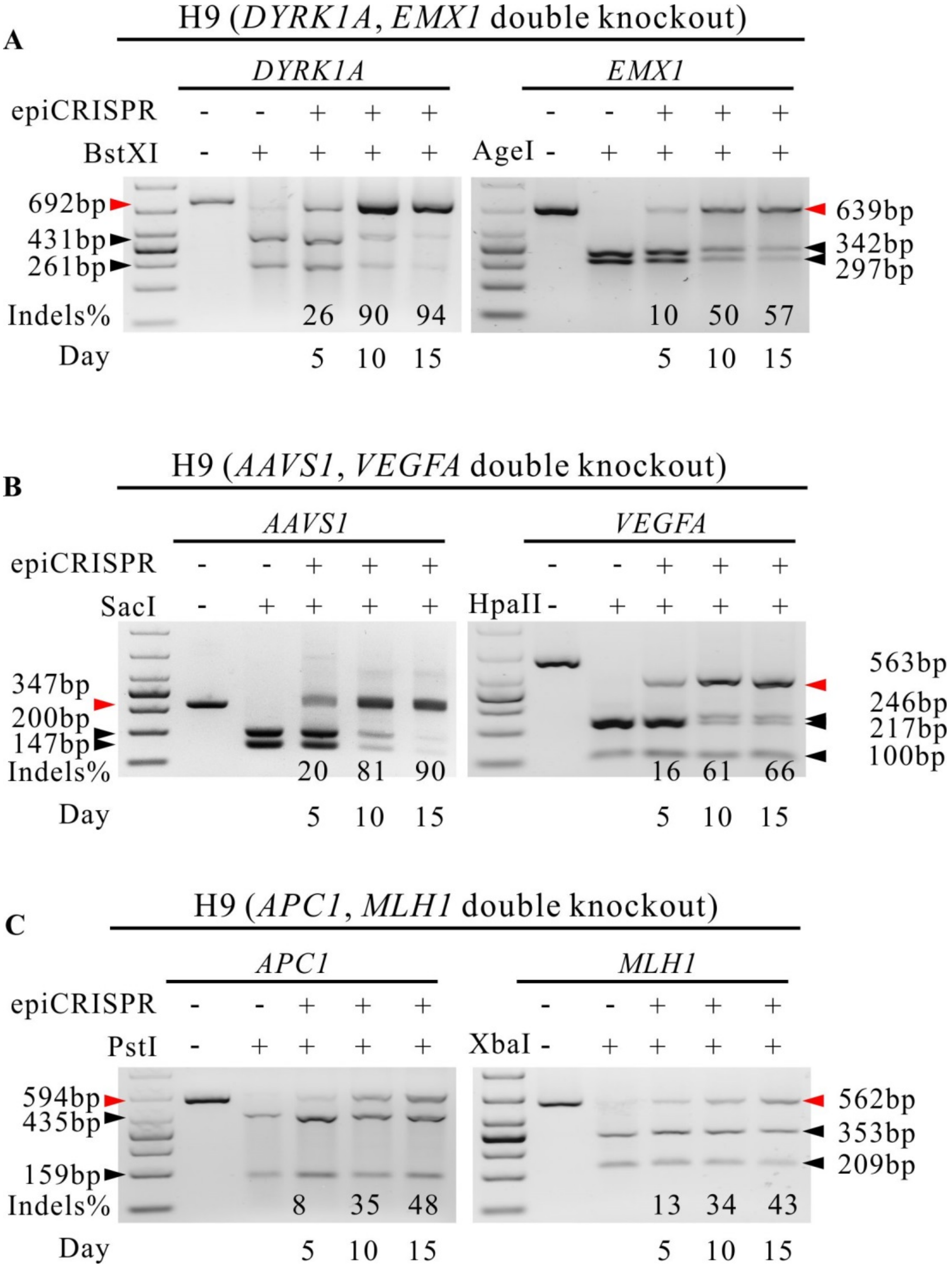

Figure S4. Representative gel pictures for RFLP analysis of double-gene knockout in hESCs. (A-C) The indel rates generated by the epiCRISPR system with gRNA multiplexed targeting *DYRK1A&EMX1*, *AAVS1&VEGFA*, and *APC1&MLH1* loci were analyzed by RFLP assay. Red triangles indicate the epiCRISPR-modified PCR bands; black triangles indicate the unmodified PCR bands.

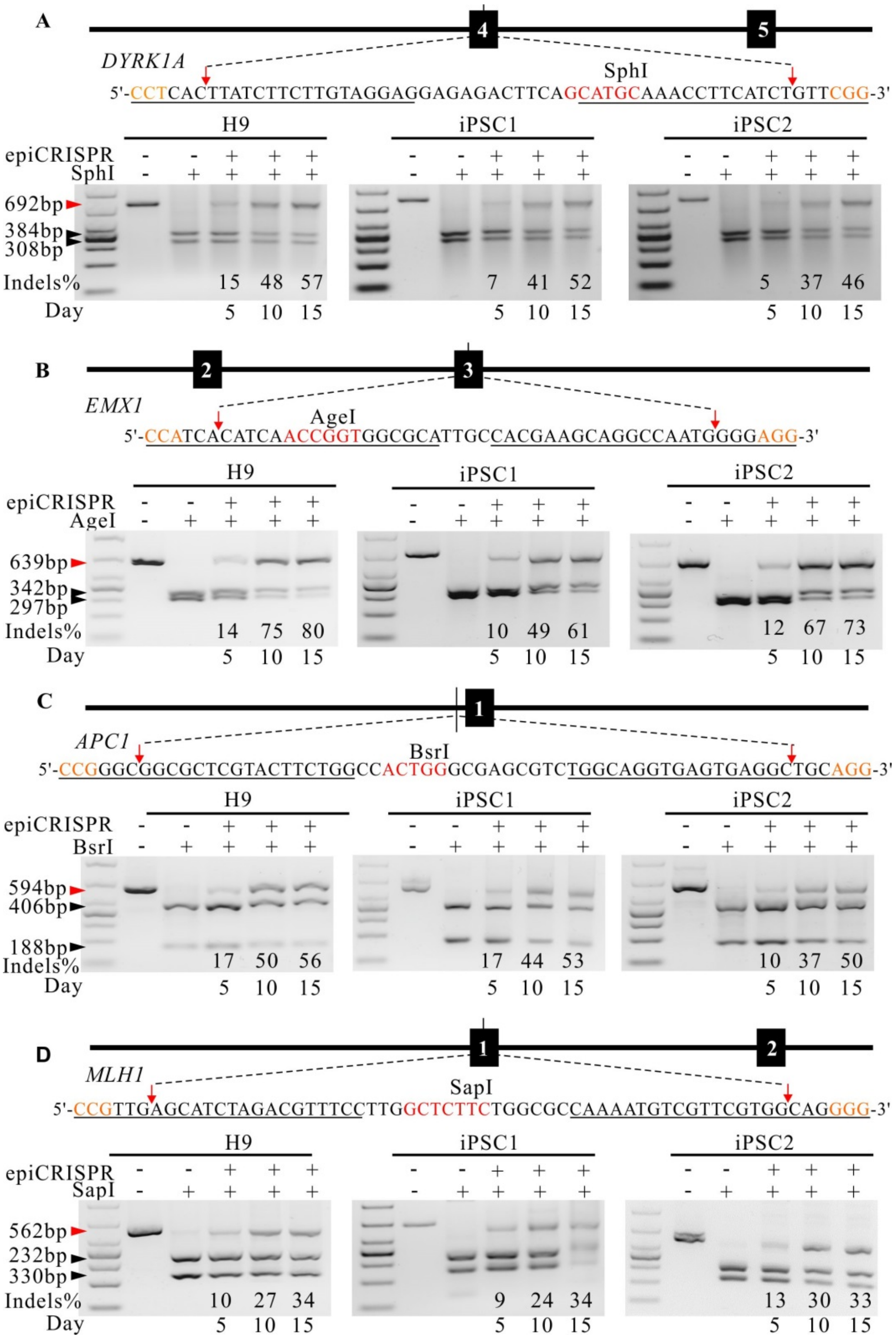

Figure S5. Representative gel pictures for RFLP analysis of indel rates generated by the epiCRISPRn for *DYRK1A*, *EMX1*, *APC1* and *MLH1* Loci in hPSCs. (A-D) RFLP analysis of indel rates at *DYRK1A* (A), *EMX1* (B), *APC1* (C) and *MLH1* (D) loci. The schematic diagrams show the gRNA targeting locus on chromosome with gRNA sequence shown below. The gRNA sequence is underlined; the restriction site is shown in red; red triangles indicate the epiCRISPR-modified PCR bands; black triangles indicate the unmodified PCR bands; red arrows indicate Cas9 cutting site. RFLP analysis was performed at day 5, 10 and 15 respectively.

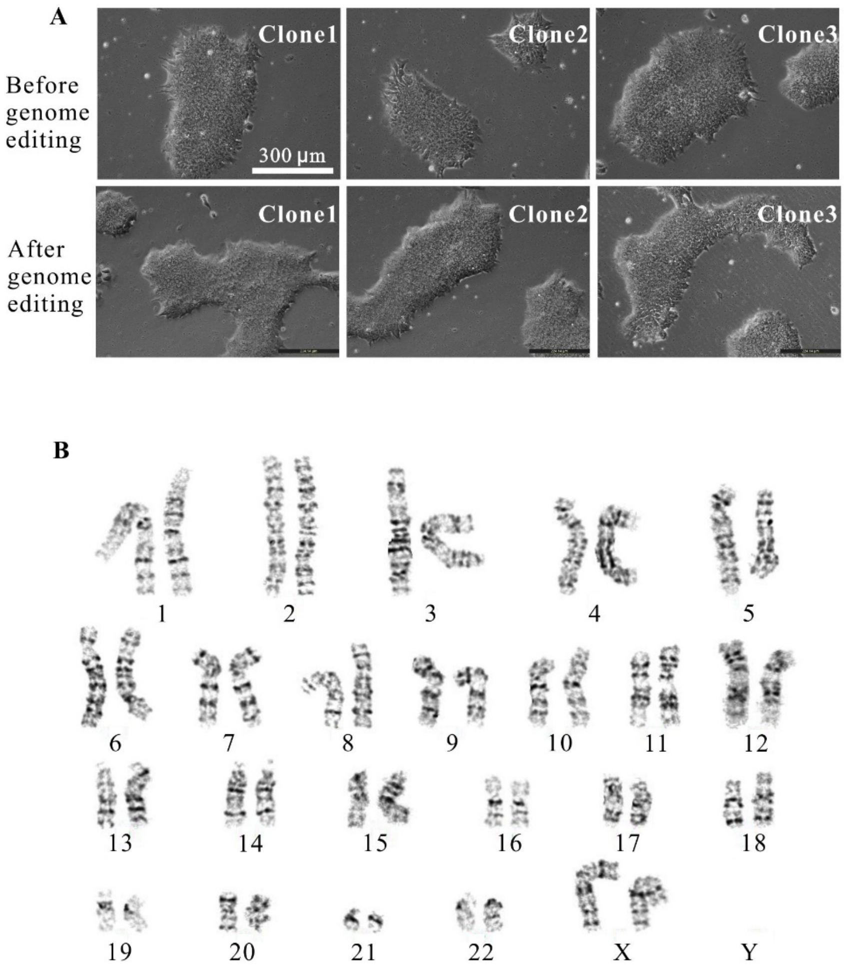

Figure S6. Morphology analysis of the epiCRISPR-modified hESCs. (A) The hESC colonies displayed normal morphology after genome editing. (B) The hESCs showed normal karyotype after genome editing.

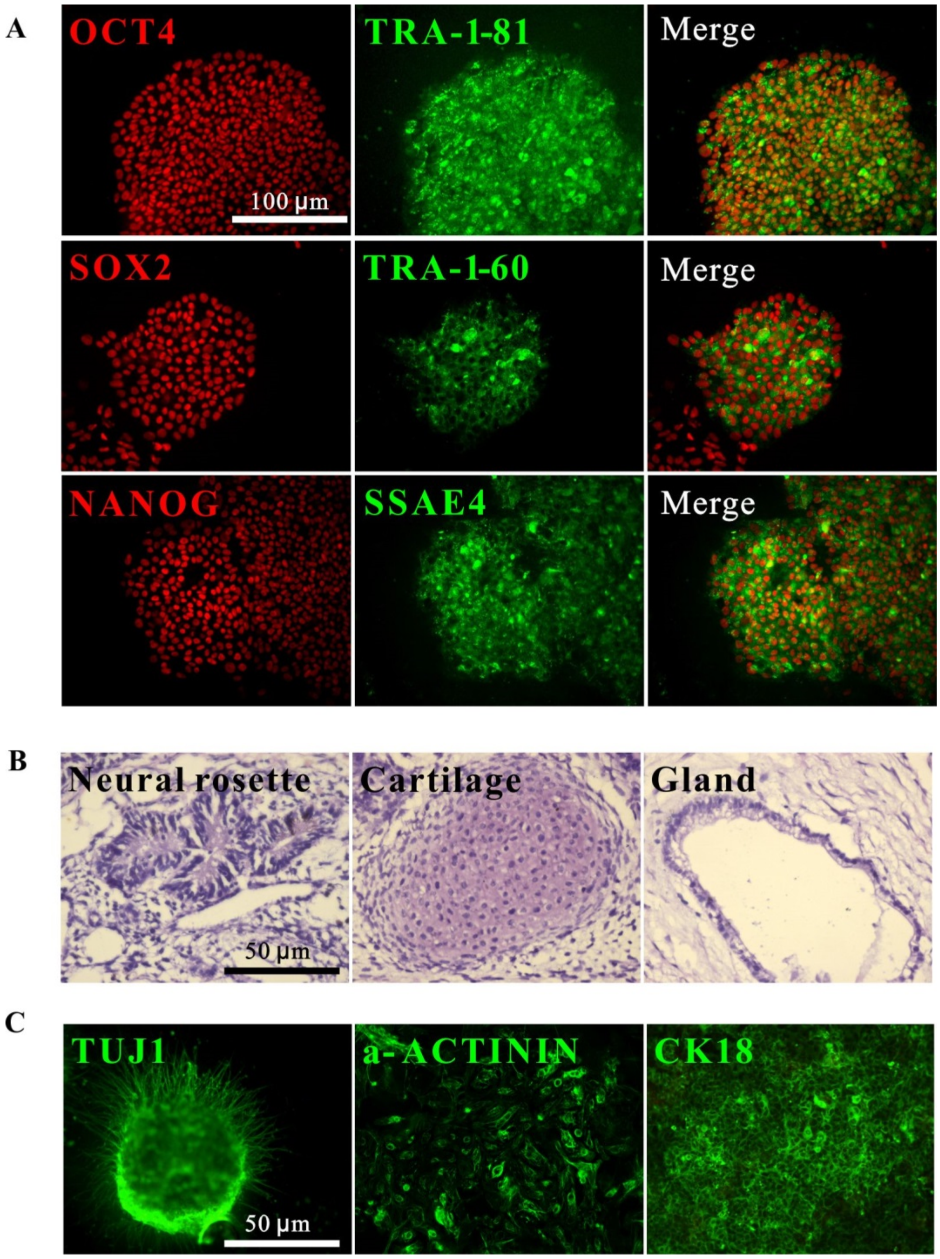

Figure S7. Pluripotency analysis of the epiCRISPR-modified hESCs. (A) The epiCRISPR-modified hESCs expressed pluripotency markers that included OCT4, TRA-1–81, SOX2, TRA-1–60, NANOG, and SSEA4. Blue stainings (right) are 4'-6-diamidino-2- phenylindole (DAPI) staining of nuclei. (B) The epiCRISPR modified hESCs formed teratomas in vivo in immunodeficient mice. The teratomas contain all 3 germ layers, identified here as neural rosette ectoderm (left), cartilage mesoderm (top right), and gland endoderm (bottom right). (C) The epiCRISPR -modified hESCs formed embryoid bodies (EBs) in vitro containing all 3 germ layer cells by expression of the neural marker TUJ1 ectoderm marker (left), smooth muscle microfilament protein (a-ACTININ) mesoderm marker (middle), and CK18 endoderm marker (right).

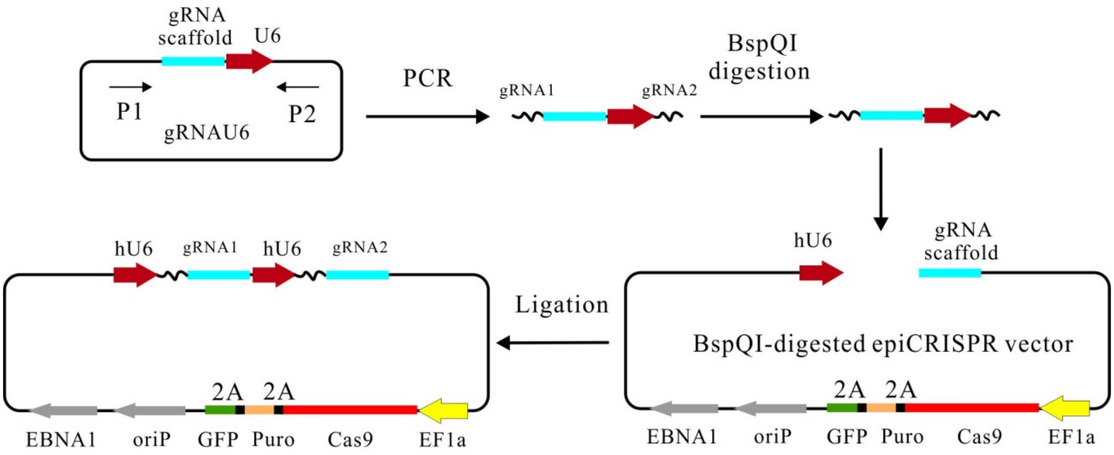

P1: CGCTCTTCGCCGNNNNN NNNNN NNNNN NNNNNgttttagagctagaaatagcaa

SapI site gRNA1

P2: CGCTCTTCTAACNNNNN NNNNN NNNNN NNNNNcggtgtttcgtcctttccac

SapI site gRNA2 (reverse complement)

Figure S8. Schematic of cloning two gRNAs into the epiCRISPR (n) vector. The primers (P1 and P2) containing gRNA sequence and BspQI restriction sequence are used to amplify tracrRNA-hU6 from an intermediate plasmid (gRNAU6). The PCR products were digested with BspQI restriction enzyme, and then subcloned into BspQI-digested epiCRISPR vector. Notably, the gRNA sequence in P2 is reverse complement.

Figure S9. DNA sequences of epiCRISPR, epiCRISPRe, epiCRISPRn and gRNAU6 plasmids.

epiCRISPR sequence (human U6 promoter colored in green, gRNA scaffold colored in blue, BspQI restriction sites underlined, EF1a promoter colored in red, SpCas9 colored in purple, P2A sequences colored in pink, Puromycin resistance gene colored in bright blue, copGFP colored in bright green, EBNA1/ OriP sequence colored in grey.

cattatggtacctttcagacccacctcccaaccccgaggggacccagagagggcctatttcccatgattccttcatatttgcat
atacgatacaaggctgttagagagataattagaattaatttgactgtaaacacaaagatattagtacaaaatacgtgacgtaga
aagtaataatttcttgggtagtttgcagttttaaaattatgttttaaaatggactatcatatgcttaccgtaacttgaaagtatttcga
tttcttggctttatatatcttgtggaaaggacgaaacaccgagaagagcgatgctcttcggttttagagctagaaatagcaagtt
aaaataaggctagtccgttatcaacttgaaaaagtggcaccgagtcggtgcttttttgaattctagatcttgagacaaatggca
gtattcatccacaattttaaaagaaaaggggggattggggggtacagtgcaggggaaagaatagtagacataatagcaac
agacatacaaactaaagaattacaaaaacaaattacaaaaattcaaaattttcgggtttattacagggacagcagagatccac
tttggcgccggctcgagtggctccggtgcccgtcagtgggcagagcgcacatcgcccacagtccccgagaagttgggggg
aggggtcggcaattgaaccggtgcctagagaaggtggcgcggggtaaactgggaaagtgatgtcgtgtactggctccg
cctttttcccgagggtgggggagaaccgtatataagtgcagtagtcgccgtgaacgttctttttcgcaacgggtttgccgcca
gaacacaggtgtcgtgacgcgggatccgccaccatggattacaaagacgatgacgataagatggccccaaagaagaag
cggaaggtcggtatccacggagtcccagcagccgacaagaagtacagcatcggcctggacatcggcaccaactctgtgg
gctgggccgtgatcaccgacgagtacaaggtgcccagcaagaaattcaaggtgctgggcaacaccgaccggcacagca
tcaagaagaacctgatcggagccctgctgttcgacagcggcgaaacagccgaggccacccggctgaagagaaccgcca
gaagaagatacaccagacggaagaaccggatctgctatctgcaagagatcttcagcaacgagatggccaaggtggacga
cagcttcttccacagactggaagagtccttcctggtggaagaggataagaagcacgagcggcaccccatcttcggcaaca
tcgtggacgaggtggcctaccacgagaagtaccccaccatctaccacctgagaaagaaactggtggacagcaccgacaa
ggccgacctgcggctgatctatctggccctggcccacatgatcaagttccggggccacttcctgatcgagggcgacctga
accccgacaacagcgacgtggacaagctgttcatccagctggtgcagacctacaaccagctgttcgaggaaaaccccatc
aacgccagcggcgtggacgccaaggccatcctgtctgccagactgagcaagagcagacggctggaaaatctgatcgcc
cagctgcccggcgagaagaagaatggcctgttcggcaacctgattgccctgagcctgggcctgacccccaacttcaaga
gcaacttcgacctggccgaggatgccaaactgcagctgagcaaggacacctacgacgacgacctggacaacctgctgg
cccagatcggcgaccagtacgccgacctgtttctggccgccaagaacctgtccgacgccatcctgctgagcgacatcctg
agagtgaacaccgagatcaccaaggcccccctgagcgcctctatgatcaagagatacgacgagcaccaccaggacctg
accctgctgaaagctctcgtgcggcagcagctgcctgagaagtacaaagagattttcttcgaccagagcaagaacggcta
cgccggctacattgacggcggagccagccaggaagagttctacaagttcatcaagcccatcctggaaaagatggacggc
accgaggaactgctcgtgaagctgaacagagaggacctgctgcggaagcagcggaccttcgacaacggcagcatcccc
caccagatccacctgggagagctgcacgccattctgcggcggcaggaagatttttacccattcctgaaggacaaccggga
aaagatcgagaagatcctgaccttccgcatcccctactacgtgggccctctggccaggggaaacagcagattcgcctgga
tgaccagaaagagcgaggaaaccatcaccccctggaacttcgaggaagtggtggacaagggcgcttccgcccagagctt
catcgagcggatgaccaacttcgataagaacctgcccaacgagaaggtgctgcccaagcacagcctgctgtacgagtact
tcaccgtgtataacgagctgaccaaagtgaaatacgtgaccgagggaatgagaaagcccgccttcctgagcggcgagca
gaaaaaggccatcgtggacctgctgttcaagaccaaccggaaagtgaccgtgaagcagctgaaagaggactacttcaag
aaaatcgagtgcttcgactccgtggaaatctccggcgtggaagatcggttcaacgcctccctgggcacataccacgatctg
ctgaaaattatcaaggacaaggacttcctggacaatgaggaaaacgaggacattctggaagatatcgtgctgaccctgaca
ctgtttgaggacagagagatgatcgaggaacggctgaaaacctatgcccacctgttcgacgacaaagtgatgaagcagct
gaagcggcggagatacaccggctggggcaggctgagccggaagctgatcaacggcatccgggacaagcagtccggc

aagacaatcctggatttcctgaagtccgacggcttcgccaacagaaacttcatgcagctgatccacgacgacagcctgacc
tttaaagaggacatccagaaagcccaggtgtccggccagggcgatagcctgcacgagcacattgccaatctggccggca
gccccgccattaagaagggcatcctgcagacagtgaaggtggtggacgagctcgtgaaagtgatgggccggcacaagc
ccgagaacatcgtgatcgaaatggccagagagaaccagaccacccagaagggacagaagaacagccgcgagagaat
gaagcggatcgaagagggcatcaaagagctgggcagccagatcctgaaagaacaccccgtggaaaacacccagctgc
agaacgagaagctgtacctgtactacctgcagaatgggcgggatatgtacgtggaccaggaactggacatcaaccggctg
tccgactacgatgtggaccatatcgtgcctcagagctttctgaaggacgactccatcgacaacaaggtgctgaccagaagc
gacaagaaccggggcaagagcgacaacgtgccctccgaagaggtcgtgaagaagatgaagaactactggcggcagct
gctgaacgccaagctgattacccagagaaagttcgacaatctgaccaaggccgagagaggcggcctgagcgaactggat
aaggccggcttcatcaagagacagctggtggaaacccggcagatcacaaagcacgtggcacagatcctggactcccgg
atgaacactaagtacgacgagaatgacaagctgatccgggaagtgaaagtgatcaccctgaagtccaagctggtgtccga
tttccggaaggatttccagttttacaaagtgcgcgagatcaacaactaccaccacgcccacgacgcctacctgaacgccgt
cgtgggaaccgccctgatcaaaaagtaccctaagctggaaagcgagttcgtgtacggcgactacaaggtgtacgacgtgc
ggaagatgatcgccaagagcgagcaggaaatcggcaaggctaccgccaagtacttcttctacagcaacatcatgaactttt
tcaagaccgagattaccctggccaacggcgagatccggaagcggcctctgatcgagacaaacggcgaaaccggggag
atcgtgtgggataagggccgggattttgccaccgtgcggaaagtgctgagcatgccccaagtgaatatcgtgaaaaagac
cgaggtgcagacaggcggcttcagcaaagagtctatcctgcccaagaggaacagcgataagctgatcgccagaaagaa
ggactgggaccctaagaagtacggcggcttcgacagccccaccgtggcctattctgtgctggtggtggccaaagtggaaa
agggcaagtccaagaaactgaagagtgtgaaagagctgctggggatcaccatcatggaaagaagcagcttcgagaaga
atcccatcgactttctggaagccaagggctacaaagaagtgaaaaaggacctgatcatcaagctgcctaagtactccctgtt
cgagctggaaaacggccggaagagaatgctggcctctgccggcgaactgcagaagggaaacgaactggccctgccctc
caaatatgtgaacttcctgtacctggccagccactatgagaagctgaagggctcccccgaggataatgagcagaaacagct
gtttgtggaacagcacaagcactacctggacgagatcatcgagcagatcagcgagttctccaagagagtgatcctggccg
acgctaatctggacaaagtgctgtccgcctacaacaagcaccgggataagcccatcagagagcaggccgagaatatcatc
cacctgtttaccctgaccaatctgggagcccctgccgccttcaagtactttgacaccaccatcgaccggaagaggtacacc
agcaccaaagaggtgctggacgccaccctgatccaccagagcatcaccggcctgtacgagacacggatcgacctgtctc
agctgggaggcgacaagcgtcctgctgctactaagaaagctggtcaagctaagaaaaagaaagctagcggcagcggcg
ccaccaacttcagcctgctgaagcaggccggcgacgtggaggagaaccccggccccatgaccgagtacaagcccacg
gtgcgcctcgccacccgcgacgacgtccccagggccgtacgcaccctcgccgccgcgttcgccgactaccccgccacg
cgccacaccgtcgatccggaccgccacatcgagcgggtcaccgagctgcaagaactcttcctcacgcgcgtcgggctcg
acatcggcaaggtgtgggtcgcggacgacggcgcggccgtggcggtctggaccacgccggagagcgtcgaagcggg
ggcggtgttcgccgagatcggcccgcgcatggccgagttgagcggttcccggctggccgcgcagcaacagatggaagg
cctcctggcgccgcaccggcccaaggagcccgcgtggttcctggccaccgtcggagtctcgcccgaccaccagggcaa
gggtctgggcagcgccgtcgtgctccccggagtggaggcggccgagcgcgccggggtgcccgccttcctggagacct
ccgcgccccgcaacctccccttctacgagcggctcggcttcaccgtcaccgccgacgtcgaggtgcccgaaggaccgc
gcacctggtgcatgacccgcaagcccggtgccgcgatcgccgaaggatccgcggccgctgagggcagaggaagtcttc
taacatgcggtgacgtggaggagaatcccggcccttccggaatggagagcgacgagagcggcctgcccgccatggaga
tcgagtgccgcatcaccggcaccctgaacggcgtggagttcgagctggtgggcggcggagagggcacccccaagcag
ggccgcatgaccaacaagatgaaaagcaccaaaggcgccctgaccttcagcccctacctgctgagccacgtgatgggct
acggcttctaccacttcggcacctaccccagcggctacgagaaccccttcctgcacgccatcaacaacggcggctacacc
aacacccgcatcgagaagtacgaggacggcggcgtgctgcacgtgagcttcagctaccgctacgaggccggccgcgtg
atcggcgacttcaaggtggtgggcaccggcttccccgaggacagcgtgatcttcaccgacaagatcatccgcagcaacg
ccaccgtggagcacctgcaccccatgggcgataacgtgctggtgggcagcttcgcccgcaccttcagcctgcgcgacgg
cggctactacagcttcgtggtggacagccacatgcacttcaagagcgccatccaccccagcatcctgcagaacgggggc

cccatgttcgccttccgccgcgtggaggagctgcacagcaacaccgagctgggcatcgtggagtaccagcacgccttca
agacccccatcgccttcgccagatcccgcgctcagtcgtccaattctgccgtggacggcaccgccggacccggctccacc
ggatctcgctagagctgaatctaagtcgacttaagaaccgctcgaggccggcaaggccggatccagacatgataagatac
attgatgagtttggacaaaccacaactagaatgcagtgaaaaaaatgctttatttgtgaaatttgtgatgctattgctttatttgta
accattataagctgcaataaacaagttaacaacaacaattgcattcattttatgtttcaggttcagggggaggtgtgggaggttt
tttaaagcaagtaaaacctctacaaatgtggtatggctgattatgatccggctgcctcgcgcgtttcggtgatgacggtgaaa
acctctgacacatgcagctcccggagacggtcacagcttgtctgtaagcggatgccgggagcagacaagcccgtcaggg
cgcgtcagcgggtgttggcgggtgtcggggcgcagccatgaggtcgactctagaggatcgatgccccgccccggacga
actaaacctgactacgacatctctgccccttcttcgcggggcagtgcatgtaatcccttcagttggttggtacaacttgccaac
tgggccctgttccacatgtgacacggggggggaccaaacacaaaggggttctctgactgtagttgacatccttataaatgga
tgtgcacatttgccaacactgagtggctttcatcctggagcagactttgcagtctgtggactgcaacacaacattgcctttatgt
gtaactcttggctgaagctcttacaccaatgctgggggacatgtacctcccaggggcccaggaagactacgggaggctac
accaacgtcaatcagaggggcctgtgtagctaccgataagcggaccctcaagagggcattagcaatagtgtttataaggcc
cccttgttaaccctaaacgggtagcatatgcttcccgggtagtagtatatactatccagactaaccctaattcaatagcatatgt
tacccaacgggaagcatatgctatcgaattagggttagtaaaagggtcctaaggaacagcgatatctcccaccccatgagc
tgtcacggttttatttacatggggtcaggattccacgagggtagtgaaccattttagtcacaagggcagtggctgaagatcaa
ggagcgggcagtgaactctcctgaatcttcgcctgcttcttcattctccttcgtttagctaatagaataactgctgagttgtgaac
agtaaggtgtatgtgaggtgctcgaaaacaaggtttcaggtgacgcccccagaataaaatttggacgggggggttcagtggt
ggcattgtgctatgacaccaatataaccctcacaaaccccttgggcaataaatactagtgtaggaatgaaacattctgaatatc
tttaacaatagaaatccatggggtggggacaagccgtaaagactggatgtccatctcacacgaatttatggctatgggcaac
acataatcctagtgcaatatgatactggggttattaagatgtgtcccaggcagggaccaagacaggtgaaccatgttgttaca
ctctatttgtaacaaggggaaagagagtggacgccgacagcagcggactccactggttgtctctaacacccccgaaaatta
aacggggctccacgccaatggggcccataaacaaagacaagtggccactcttttttttgaaattgtggagtgggggcacgc
gtcagcccccacacgccgccctgcggttttggactgtaaaataagggtgtaataacttggctgattgtaaccccgctaacca
ctgcggtcaaaccacttgcccacaaaaccactaatggcaccccggggaatacctgcataagtaggtgggcgggccaaga
taggggcgcgattgctgcgatctggaggacaaattacacacacttgcgcctgagcgccaagcacagggttgttggtcctca
tattcacgaggtcgctgagagcacggtgggctaatgttgccatgggtagcatatactacccaaatatctggatagcatatgct
atcctaatctatatctgggtagcataggctatcctaatctatatctgggtagcatatgctatcctaatctatatctgggtagtatat
gctatcctaatttatatctgggtagcataggctatcctaatctatatctgggtagcatatgctatcctaatctatatctgggtagtat
atgctatcctaatctgtatccgggtagcatatgctatcctaatagagattagggtagtatatgctatcctaatttatatctgggtag
catatactacccaaatatctggatagcatatgctatcctaatctatatctgggtagcatatgctatcctaatctatatctgggtagc
ataggctatcctaatctatatctgggtagcatatgctatcctaatctatatctgggtagtatatgctatcctaatttatatctgggta
gcataggctatcctaatctatatctgggtagcatatgctatcctaatctatatctgggtagtatatgctatcctaatctgtatccgg
gtagcatatgctatcctcatgcatatacagtcagcatatgatacccagtagtagagtgggagtgctatcctttgcatatgccgc
cacctcccaagggggcgtgaattttcgctgcttgtccttttcctgctggttgctcccattcttaggtgaatttaaggaggccagg
ctaaagccgtcgcatgtctgattgctcaccaggtaaatgtcgctaatgttttccaacgcgagaaggtgttgagcgcggagct
gagtgacgtgacaacatgggtatgcccaattgccccatgttgggaggacgaaaatggtgacaagacagatggccagaaat
acaccaacagcacgcatgatgtctactggggatttattctttagtgcgggggaatacacggcttttaatacgattgagggcgt
ctcctaacaagttacatcactcctgcccttcctcaccctcatctccatcacctccttcatctccgtcatctccgtcatcaccctcc
gcggcagccccttccaccataggtggaaaccagggaggcaaatctactccatcgtcaaagctgcacacagtcaccctgat
attgcaggtaggagcgggctttgtcataacaaggtccttaatcgcatccttcaaaacctcagcaaatatatgagtttgtaaaaa
gaccatgaaataacagacaatggactcccttagcgggccaggttgtgggccgggtccaggggccattccaaaggggaga
cgactcaatggtgtaagacgacattgtggaatagcaagggcagttcctcgccttaggttgtaaagggaggtcttactacctc
catatacgaacacaccggcgacccaagttccttcgtcggtagtcctttctacgtgactcctagccaggagagctcttaaacct

tctgcaatgttctcaaatttcgggttggaacctccttgaccacgatgctttccaaaccaccctcctttttgcgcctgcctccatc
accctgaccccggggtccagtgcttgggccttctcctgggtcatctgcggggccctgctctatcgctcccgggggcacgtc
aggctcaccatctgggccaccttcttggtggtattcaaaataatcggcttcccctacagggtggaaaaatggccttctacctg
gagggggcctgcgcggtggagacccggatgatgatgactgactactgggactcctgggcctcttttctccacgtccacga
cctctccccctggctctttcacgacttccccccctggctctttcacgtcctctaccccggcggcctccactacctcctcgaccc
cggcctccactacctcctcgaccccggcctccactgcctcctcgaccccggcctccacctcctgctcctgcccctcctgctc
ctgcccctcctcctgctcctgcccctcctgcccctcctgctcctgcccctcctgcccctcctgctcctgcccctcctgcccctc
ctgctcctgcccctcctgcccctcctcctgctcctgcccctcctgcccctcctcctgctcctgcccctcctgcccctcctgctc
ctgcccctcctgcccctcctgctcctgcccctcctgcccctcctgctcctgcccctcctgctcctgcccctcctgctcctgccc
ctcctgctcctgcccctcctgcccctcctgcccctcctcctgctcctgcccctcctgctcctgcccctcctgcccctcctgccc
ctcctgctcctgcccctcctcctgctcctgcccctcctgcccctcctgcccctcctcctgctcctgcccctcctgcccctcctcc
tgctcctgcccctcctcctgctcctgcccctcctgcccctcctgcccctcctcctgctcctgcccctcctgcccctcctcctgct
cctgcccctcctcctgctcctgcccctcctgcccctcctgcccctcctcctgctcctgcccctcctcctgctcctgcccctcct
gcccctcctgcccctcctgcccctcctcctgctcctgcccctcctcctgctcctgcccctcctgctcctgcccctcccgctcct
gctcctgctcctgttccaccgtgggtccctttgcagccaatgcaacttggacgtttttggggtctccggacaccatctctatgtc
ttggccctgatcctgagccgcccggggctcctggtcttccgcctcctcgtcctcgtcctcttccccgtcctcgtccatggttat
cacccccctcttctttgaggtccactgccgccggagccttctggtccagatgtgtctcccttctctcctaggccatttccaggtcc
tgtacctggcccctcgtcagacatgattcacactaaaagagatcaatagacatctttattagacgacgctcagtgaatacagg
gagtgcagactcctgccccctccaacagcccccccaccctcatccccttcatggtcgctgtcagacagatccaggtctgaa
aattccccatcctccgaaccatcctcgtcctcatcaccaattactcgcagcccggaaaactcccgctgaacatcctcaagatt
tgcgtcctgagcctcaagccaggcctcaaattcctcgtccccctttttgctggacggtagggatggggattctcgggacccc
tcctcttcctcttcaaggtcaccagacagagatgctactggggcaacggaagaaaagctgggtgcggcctgtgaggatca
gcttatcgatgataagctgtcaaacatgagaattcttgaagacgaaagggcctcgtgatacgcctatttttataggttaatgtca
tgataataatggtttcttagacgtcaggtggcacttttcggggaaatgtgcgcggaacccctatttgtttatttttctaaatacatt
caaatatgtatccgctcatgagacaataaccctgataaatgcttcaataatattgaaaaaggaagagtatgagtattcaacattt
ccgtgtcgcccttattcccttttttgcggcattttgccttcctgtttttgctcacccagaaacgctggtgaaagtaaaagatgctg
aagatcagttgggtgcacgagtgggttacatcgaactggatctcaacagcggtaagatccttgagagttttcgccccgaaga
acgttttccaatgatgagcacttttaaagttctgctatgtggcgcggtattatcccgtgttgacgccgggcaagagcaactcgg
tcgccgcatacactattctcagaatgacttggttgagtactcaccagtcacagaaaagcatcttacggatggcatgacagtaa
gagaattatgcagtgctgccataaccatgagtgataacactgcggccaacttacttctgacaacgatcggaggaccgaagg
agctaaccgcttttttgcacaacatgggggatcatgtaactcgccttgatcgttgggaaccggagctgaatgaagccatacc
aaacgacgagcgtgacaccacgatgcctgcagcaatggcaacaacgttgcgcaaactattaactggcgaactacttactct
agcttcccggcaacaattaatagactggatggaggcggataaagttgcaggaccacttctgcgctcggcccttccggctgg
ctggtttattgctgataaatctggagccggtgagcgtgggtctcgcggtatcattgcagcactggggccagatggtaagccc
tcccgtatcgtagttatctacacgacggggagtcaggcaactatggatgaacgaaatagacagatcgctgagataggtgcc
tcactgattaagcattggtaactgtcagaccaagtttactcatatatactttagattgatttaaaacttcatttttaatttaaaaggat
ctaggtgaagatcctttttgataatctcatgaccaaaatcccttaacgtgagttttcgttccactgagcgtcagaccccgtagaa
aagatcaaaggatcttcttgagatcctttttttctgcgcgtaatctgctgcttgcaaacaaaaaaaccaccgctaccagcggtg
gtttgtttgccggatcaagagctaccaactctttttccgaaggtaactggcttcagcagagcgcagataccaaatactgtcctt
ctagtgtagccgtagttaggccaccacttcaagaactctgtagcaccgcctacatacctcgctctgctaatcctgttaccagtg
gctgctgccagtggcgataagtcgtgtcttaccgggttggactcaagacgatagttaccggataaggcgcagcggtcggg
ctgaacggggggttcgtgcacacagcccagcttggagcgaacgacctacaccgaactgagatacctacagcgtgagcta
tgagaaagcgccacgcttcccgaagggagaaaggcggacaggtatccggtaagcggcagggtcggaacaggagagc
gcacgagggagcttccagggggaaacgcctggtatctttatagtcctgtcgggtttcgccacctctgacttgagcgtcgattt

ttgtgatgctcgtcaggggggcggagcctatggaaaaacgccagcaacgcggcctttttacggttcctggccttttgctggc
cttgaagctgtccctgatggtcgtcatctacctgcctggacagcatggcctgcaacgcgggcatcccgatgccgccggaa
gcgagaagaatcataatggggaaggccatccagcctcgcgtcgaactagatgatccggctgtggaatgtgtgtcagttag
ggtgtggaaagtccccaggctccccagcaggcagaagtatgcaaagcatg

epiCRISPRe sequence (mouse U6 promoter colored in green, gRNA scaffold colored in blue, BspQI restriction sites underlined, EF1a promoter colored in red, SpCas9 colored in purple, K848A/K1003A/R1060A mutations colored in yellow, P2A sequences colored in pink, Puromycin resistance gene colored in bright blue, copGFP colored in bright green, EBNA1/ OriP sequence colored in grey.

cattatgtctagagatccgacgccgccatctctaggcccgcgccggccccctcgcacagacttgtgggagaagctcggct
actcccctgccccggttaatttgcatataatatttcctagtaactatagaggcttaatgtgcgataaaagacagataatctgttctt
tttaatactagctacattttacatgataggcttggatttctataagagatacaaatactaaattattattttaaaaaacagcacaaaa
ggaaactcaccctaactgtaaagtaattgtgtgttttgagactataaatatgcatgcgagaaaagccttgtttagaagagcgat
gctcttcggttttagagctagaaatagcaagttaaaataaggctagtccgttatcaacttgaaaaagtggcaccgagtcggtg
ctttttgaattctagatcttgagacaaatggcagtattcatccacaattttaaaagaaaaggggggattggggggtacagtgc
aggggaaagaatagtagacataatagcaacagacatacaaactaaagaattacaaaaacaaattacaaaaattcaaaatttt
cgggtttattacagggacagcagagatccactttggcgccggctcgagtggctccggtgcccgtcagtgggcagagcgc
acatcgcccacagtccccgagaagttggggggaggggtcggcaattgaaccggtgcctagagaaggtggcgcggggta
aactgggaaagtgatgtcgtgtactggctccgcctttttcccgagggtgggggagaaccgtatataagtgcagtagtcgcc
gtgaacgttctttttcgcaacgggtttgccgccagaacacaggtgtcgtgacgcgggatccgccaccatggattacaaaga
cgatgacgataagatggccccaaagaagaagcggaaggtcggtatccacggagtcccagcagccgacaagaagtaca
gcatcggcctggacatcggcaccaactctgtgggctgggccgtgatcaccgacgagtacaaggtgcccagcaagaaatt
caaggtgctgggcaacaccgaccggcacagcatcaagaagaacctgatcggagccctgctgttcgacagcggcgaaac
agccgaggccacccggctgaagagaaccgccagaagaagatacaccagacggaagaaccggatctgctatctgcaag
agatcttcagcaacgagatggccaaggtggacgacagcttcttccacagactggaagagtccttcctggtggaagaggat
aagaagcacgagcggcaccccatcttcggcaacatcgtggacgaggtggcctaccacgagaagtaccccaccatctacc
acctgagaaagaaactggtggacagcaccgacaaggccgacctgcggctgatctatctggccctggcccacatgatcaa
gttccggggccacttcctgatcgagggcgacctgaaccccgacaacagcgacgtggacaagctgttcatccagctggtgc
agacctacaaccagctgttcgaggaaaaccccatcaacgccagcggcgtggacgccaaggccatcctgtctgccagact
gagcaagagcagacggctggaaaatctgatcgcccagctgcccggcgagaagaagaatggcctgttcggcaacctgatt
gccctgagcctgggcctgacccccaacttcaagagcaacttcgacctggccgaggatgccaaactgcagctgagcaagg
acacctacgacgacgacctggacaacctgctggcccagatcggcgaccagtacgccgacctgtttctggccgccaagaa
cctgtccgacgccatcctgctgagcgacatcctgagagtgaacaccgagatcaccaaggcccccctgagcgcctctatga
tcaagagatacgacgagcaccaccaggacctgaccctgctgaaagctctcgtgcggcagcagctgcctgagaagtacaa
agagattttcttcgaccagagcaagaacggctacgccggctacattgacggcggagccagccaggaagagttctacaagt
tcatcaagcccatcctggaaaagatggacggcaccgaggaactgctcgtgaagctgaacagagaggacctgctgcggaa
gcagcggaccttcgacaacggcagcatcccccaccagatccacctgggagagctgcacgccattctgcggcggcagga
agatttttacccattcctgaaggacaaccgggaaaagatcgagaagatcctgaccttccgcatcccctactacgtgggccct
ctggccaggggaaacagcagattcgcctggatgaccagaaagagcgaggaaaccatcacccccctggaacttcgaggaa
gtggtggacaagggcgcttccgcccagagcttcatcgagcggatgaccaacttcgataagaacctgcccaacgagaagg
tgctgcccaagcacagcctgctgtacgagtacttcaccgtgtataacgagctgaccaaagtgaaatacgtgaccgaggga
atgagaaagcccgccttcctgagcggcgagcagaaaaaggccatcgtggacctgctgttcaagaccaaccggaaagtga

ccgtgaagcagctgaaagaggactacttcaagaaaatcgagtgcttcgactccgtggaaatctccggcgtggaagatcgg
ttcaacgcctccctgggcacataccacgatctgctgaaaattatcaaggacaaggacttcctggacaatgaggaaaacgag
gacattctggaagatatcgtgctgaccctgacactgtttgaggacagagagatgatcgaggaacggctgaaaacctatgcc
cacctgttcgacgacaaagtgatgaagcagctgaagcggcggagatacaccggctggggcaggctgagccggaagctg
atcaacggcatccgggacaagcagtccggcaagacaatcctggatttcctgaagtccgacggcttcgccaacagaaactt
catgcagctgatccacgacgacagcctgacctttaaagaggacatccagaaagcccaggtgtccggccagggcgatagc
ctgcacgagcacattgccaatctggccggcagccccgccattaagaagggcatcctgcagacagtgaaggtggtggacg
agctcgtgaaagtgatgggccggcacaagcccgagaacatcgtgatcgaaatggccagagagaaccagaccacccaga
agggacagaagaacagccgcgagagaatgaagcggatcgaagagggcatcaaagagctgggcagccagatcctgaa
agaacaccccgtggaaaacacccagctgcagaacgagaagctgtacctgtactacctgcagaatgggcgggatatgtac
gtggaccaggaactggacatcaaccggctgtccgactacgatgtggaccatatcgtgcctcagagctttctggcggacga
ctccatcgacaacaaggtgctgaccagaagcgacaagaaccggggcaagagcgacaacgtgccctccgaagaggtcgt
gaagaagatgaagaactactggcggcagctgctgaacgccaagctgattacccagagaaagttcgacaatctgaccaag
gccgagagaggcggcctgagcgaactggataaggccggcttcatcaagagacagctggtggaaacccggcagatcac
aaagcacgtggcacagatcctggactcccggatgaacactaagtacgacgagaatgacaagctgatccgggaagtgaaa
gtgatcaccctgaagtccaagctggtgtccgatttccggaaggatttccagttttacaaagtgcgcgagatcaacaactacca
ccacgcccacgacgcctacctgaacgccgtcgtgggaaccgccctgatcaaaaagtaccctgcgctggaaagcgagttc
gtgtacggcgactacaaggtgtacgacgtgcggaagatgatcgccaagagcgagcaggaaatcggcaaggctaccgcc
aagtacttcttctacagcaacatcatgaactttttcaagaccgagattaccctggccaacggcgagatccggaaggcgcctc
tgatcgagacaaacggcgaaaccggggagatcgtgtgggataagggccgggattttgccaccgtgcggaaagtgctga
gcatgccccaagtgaatatcgtgaaaaagaccgaggtgcagacaggcggcttcagcaaagagtctatcctgcccaagag
gaacagcgataagctgatcgccagaaagaaggactgggaccctaagaagtacggcggcttcgacagccccaccgtggc
ctattctgtgctggtggtggccaaagtggaaaagggcaagtccaagaaactgaagagtgtgaaagagctgctggggatca
ccatcatggaaagaagcagcttcgagaagaatcccatcgactttctggaagccaagggctacaaagaagtgaaaaagga
cctgatcatcaagctgcctaagtactccctgttcgagctggaaaacggccggaagagaatgctggcctctgccggcgaact
gcagaagggaaacgaactggccctgccctccaaatatgtgaacttcctgtacctggccagccactatgagaagctgaagg
gctcccccgaggataatgagcagaaacagctgtttgtggaacagcacaagcactacctggacgagatcatcgagcagatc
agcgagttctccaagagagtgatcctggccgacgctaatctggacaaagtgctgtccgcctacaacaagcaccgggataa
gcccatcagagagcaggccgagaatatcatccacctgtttaccctgaccaatctgggagcccctgccgccttcaagtacttt
gacaccaccatcgaccggaagaggtacaccagcaccaaagaggtgctggacgccaccctgatccaccagagcatcacc
ggcctgtacgagacacggatcgacctgtctcagctgggaggcgacaagcgtcctgctgctactaagaaagctggtcaagc
taagaaaaagaaagctagcggcagcggcgccaccaacttcagcctgctgaagcaggccggcgacgtggaggagaacc
ccggccccatgaccgagtacaagcccacggtgcgcctcgccacccgcgacgacgtccccagggccgtacgcaccctc
gccgccgcgttcgccgactaccccgccacgcgccacaccgtcgatccggaccgccacatcgagcgggtcaccgagctg
caagaactcttcctcacgcgcgtcgggctcgacatcggcaaggtgtgggtcgcggacgacggcgcggccgtggcggtct
ggaccacgccggagagcgtcgaagcgggggcggtgttcgccgagatcggcccgcgcatggccgagttgagcggttcc
cggctggccgcgcagcaacagatggaaggcctcctggcgccgcaccggcccaaggagcccgcgtggttcctggccac
cgtcggagtctcgcccgaccaccagggcaagggtctgggcagcgccgtcgtgctccccggagtggaggcggccgagc
gcgccggggtgcccgccttcctggagacctccgcgccccgcaacctccccttctacgagcggctcggcttcaccgtcacc
gccgacgtcgaggtgcccgaaggaccgcgcacctggtgcatgacccgcaagcccggtgccgcgatcgccgaaggatc
cgcggccgctgagggcagaggaagtcttctaacatgcggtgacgtggaggagaatcccggcccttccggaatggagag
cgacgagagcggcctgcccgccatggagatcgagtgccgcatcaccggcaccctgaacggcgtggagttcgagctggt
gggcggcggagagggcacccccaagcagggccgcatgaccaacaagatgaaaagcaccaaaggcgccctgaccttc
agcccctacctgctgagccacgtgatgggctacggcttctaccacttcggcacctaccccagcggctacgagaacccttc

ctgcacgccatcaacaacggcggctacaccaacacccgcatcgagaagtacgaggacggcggcgtgctgcacgtgagc
ttcagctaccgctacgaggccggccgcgtgatcggcgacttcaaggtggtgggcaccggcttccccgaggacagcgtga
tcttcaccgacaagatcatccgcagcaacgccaccgtggagcacctgcaccccatgggcgataacgtgctggtgggcag
cttcgcccgcaccttcagcctgcgcgacggcggctactacagcttcgtggtggacagccacatgcacttcaagagcgcca
tccaccccagcatcctgcagaacgggggccccatgttcgccttccgccgcgtggaggagctgcacagcaacaccgagct
gggcatcgtggagtaccagcacgccttcaagacccccatcgccttcgccagatcccgcgctcagtcgtccaattctgccgt
ggacggcaccgccggacccggctccaccggatctcgctagagctgaatctaagtcgacttaagaaccgctcgaggccgg
caaggccggatccagacatgataagatacattgatgagtttggacaaaccacaactagaatgcagtgaaaaaaatgctttat
ttgtgaaatttgtgatgctattgctttatttgtaaccattataagctgcaataaacaagttaacaacaacaattgcattcattttatgt
ttcaggttcagggggaggtgtgggaggttttttaaagcaagtaaaacctctacaaatgtggtatggctgattatgatccggct
gcctcgcgcgtttcggtgatgacggtgaaaacctctgacacatgcagctcccggagacggtcacagcttgtctgtaagcgg
atgccgggagcagacaagcccgtcagggcgcgtcagcgggtgttggcgggtgtcggggcgcagccatgaggtcgact
ctagaggatcgatgccccgccccggacgaactaaacctgactacgacatctctgccccttcttcgcggggcagtgcatgta
atcccttcagttggttggtacaacttgccaactgggccctgttccacatgtgacacggggggggaccaaacacaaaggggt
tctctgactgtagttgacatccttataaatggatgtgcacatttgccaacactgagtggctttcatcctggagcagactttgcagt
ctgtggactgcaacacaacattgcctttatgtgtaactcttggctgaagctcttacaccaatgctgggggacatgtacctccca
ggggcccaggaagactacgggaggctacaccaacgtcaatcagaggggcctgtgtagctaccgataagcggaccctca
agagggcattagcaatagtgtttataaggccccc ttgttaaccctaaacgggtagcatatgcttcccgggtagtagtatatact
atccagactaaccctaattcaatagcatatgttacccaacgggaagcatatgctatcgaattagggttagtaaaagggtccta
aggaacagcgatatctcccaccccatgagctgtcacggttttatttacatggggtcaggattccacgagggtagtgaaccatt
ttagtcacaagggcagtggctgaagatcaaggagcgggcagtgaactctcctgaatcttcgcctgcttcttcattctccttcgt
ttagctaatagaataactgctgagttgtgaacagtaaggtgtatgtgaggtgctcgaaaacaaggtttcaggtgacgccccc
agaataaaatttggacgggggggttcagtggtggcattgtgctatgacaccaatataaccctcacaaaccccttgggcaataa
atactagtgtaggaatgaaacattctgaatatctttaacaatagaaatccatggggtggggacaagccgtaaagactggatgt
ccatctcacacgaatttatggctatgggcaacacataatcctagtgcaatatgatactggggttattaagatgtgtcccaggca
gggaccaagacaggtgaaccatgttgttacactctatttgtaacaaggggaaagagagtggacgccgacagcagcggact
ccactggttgtctctaacacccccgaaaattaaacggggctccacgccaatggggcccataaacaaagacaagtggccac
tctttttttgaaattgtggagtgggggcacgcgtcagcccccacacgccgccctgcggttttggactgtaaaataagggtgt
aataacttggctgattgtaaccccgctaaccactgcggtcaaaccacttgcccacaaaaccactaatggcaccccggggaa
tacctgcataagtaggtgggcgggccaagataggggcgcgattgctgcgatctggaggacaaattacacacacttgcgcc
tgagcgccaagcacagggttgttggtcctcatattcacgaggtcgctgagagcacggtgggctaatgttgccatgggtagc
atatactacccaaatatctggatagcatatgctatcctaatctatatctgggtagcataggctatcctaatctatatctgggtagc
atatgctatcctaatctatatctgggtagtatatgctatcctaatttatatctgggtagcataggctatcctaatctatatctgggta
gcatatgctatcctaatctatatctgggtagtatatgctatcctaatctgtatccgggtagcatatgctatcctaatagagattagg
gtagtatatgctatcctaatttatatctgggtagcatatactacccaaatatctggatagcatatgctatcctaatctatatctgggt
agcatatgctatcctaatctatatctgggtagcataggctatcctaatctatatctgggtagcatatgctatcctaatctatatctg
ggtagtatatgctatcctaatttatatctgggtagcataggctatcctaatctatatctgggtagcatatgctatcctaatctatatc
tgggtagtatatgctatcctaatctgtatccgggtagcatatgctatcctcatgcatatacagtcagcatatgatacccagtagt
agagtgggagtgctatcctttgcatatgccgccacctcccaagggggcgtgaattttcgctgcttgtccttttcctgctggttgc
tcccattcttaggtgaatttaaggaggccaggctaaagccgtcgcatgtctgattgctcaccaggtaaatgtcgctaatgttttc
caacgcgagaaggtgttgagcgcggagctgagtgacgtgacaacatgggtatgcccaattgccccatgttgggaggacg
aaaatggtgacaagacagatggccagaaatacaccaacagcacgcatgatgtctactggggatttattctttagtgcgggg
gaatacacggcttttaatacgattgagggcgtctcctaacaagttacatcactcctgcccttcctcaccctcatctccatcacct
ccttcatctccgtcatctccgtcatcaccctccgcggcagccccttccaccataggtggaaaccagggaggcaaatctactc

catcgtcaaagctgcacacagtcaccctgatattgcaggtaggagcgggctttgtcataacaaggtccttaatcgcatccttc
aaaacctcagcaaatatatgagtttgtaaaaagaccatgaaataacagacaatggactcccttagcgggccaggttgtggg
ccgggtccaggggccattccaaaggggagacgactcaatggtgtaagacgacattgtggaatagcaagggcagttcctc
gccttaggttgtaaagggaggtcttactacctccatatacgaacacaccggcgacccaagttccttcgtcggtagtcctttcta
cgtgactcctagccaggagagctcttaaaccttctgcaatgttctcaaatttcgggttggaacctccttgaccacgatgctttcc
aaaccaccctcctttttgcgcctgcctccatcaccctgaccccggggtccagtgcttgggccttctcctgggtcatctgcgg
ggccctgctctatcgctcccgggggcacgtcaggctcaccatctgggccaccttcttggtggtattcaaaataatcggcttcc
cctacagggtggaaaaatggccttctacctggagggggcctgcgcggtggagacccggatgatgatgactgactactgg
gactcctgggcctcttttctccacgtccacgacctctccccctggctctttcacgacttccccccctggctctttcacgtcctcta
ccccggcggcctccactacctcctcgaccccggcctccactacctcctcgaccccggcctccactgcctcctcgaccccg
gcctccacctcctgctcctgcccctcctgctcctgcccctcctcctgctcctgcccctcctgcccctcctgctcctgcccctcc
tgcccctcctgctcctgcccctcctgcccctcctgctcctgcccctcctgcccctcctcctgctcctgcccctcctgcccctcc
tcctgctcctgcccctcctgcccctcctgctcctgcccctcctgcccctcctgctcctgcccctcctgcccctcctgctcctgc
ccctcctgctcctgcccctcctgctcctgcccctcctgctcctgcccctcctgcccctcctgcccctcctcctgctcctgcccc
tcctgctcctgcccctcctgcccctcctgcccctcctgctcctgcccctcctcctgctcctgcccctcctgcccctcctgcccc
tcctcctgctcctgcccctcctgcccctcctcctgctcctgcccctcctcctgctcctgcccctcctgcccctcctgcccctcct
cctgctcctgcccctcctgcccctcctcctgctcctgcccctcctcctgctcctgcccctcctgcccctcctgcccctcctcct
gctcctgcccctcctcctgctcctgcccctcctgcccctcctgcccctcctgcccctcctcctgctcctgcccctcctcctgct
cctgcccctcctgctcctgcccctcccgctcctgctcctgctcctgttccaccgtgggtccctttgcagccaatgcaacttgga
cgtttttggggtctccggacaccatctctatgtcttggccctgatcctgagccgcccggggctcctggtcttccgcctcctcgt
cctcgtcctcttccccgtcctcgtccatggttatcacccccctcttctttgaggtccactgccgccggagccttctggtccagatg
tgtctcccttctctcctaggccatttccaggtcctgtacctggcccctcgtcagacatgattcacactaaaagagatcaataga
catctttattagacgacgctcagtgaatacagggagtgcagactcctgccccctccaacagcccccccaccctcatcccctt
catggtcgctgtcagacagatccaggtctgaaaattccccatcctccgaaccatcctcgtcctcatcaccaattactcgcagc
ccggaaaactcccgctgaacatcctcaagatttgcgtcctgagcctcaagccaggcctcaaattcctcgtcccccttttttgct
ggacggtagggatggggattctcgggacccctcctcttcctcttcaaggtcaccagacagagatgctactggggcaacgg
aagaaaagctgggtgcggcctgtgaggatcagcttatcgatgataagctgtcaaacatgagaattcttgaagacgaaaggg
cctcgtgatacgcctatttttataggttaatgtcatgataataatggtttcttagacgtcaggtggcacttttcggggaaatgtgc
gcggaacccctatttgtttatttttctaaatacattcaaatatgtatccgctcatgagacaataaccctgataaatgcttcaataat
attgaaaaaggaagagtatgagtattcaacatttccgtgtcgcccttattcccttttttgcggcattttgccttcctgtttttgctcac
ccagaaacgctggtgaaagtaaaagatgctgaagatcagttgggtgcacgagtgggttacatcgaactggatctcaacag
cggtaagatccttgagagttttcgccccgaagaacgttttccaatgatgagcacttttaaagttctgctatgtggcgcggtatta
tcccgtgttgacgccgggcaagagcaactcggtcgccgcatacactattctcagaatgacttggttgagtactcaccagtca
cagaaaagcatcttacggatggcatgacagtaagagaattatgcagtgctgccataaccatgagtgataacactgcggcca
acttacttctgacaacgatcggaggaccgaaggagctaaccgcttttttgcacaacatgggggatcatgtaactcgccttgat
cgttgggaaccggagctgaatgaagccataccaaacgacgagcgtgacaccacgatgcctgcagcaatggcaacaacg
ttgcgcaaactattaactggcgaactacttactctagcttcccggcaacaattaatagactggatggaggcggataaagttgc
aggaccacttctgcgctcggcccttccggctggctggtttattgctgataaatctggagccggtgagcgtgggtctcgcggt
atcattgcagcactggggccagatggtaagccctcccgtatcgtagttatctacacgacggggagtcaggcaactatggat
gaacgaaatagacagatcgctgagataggtgcctcactgattaagcattggtaactgtcagaccaagtttactcatatatactt
tagattgatttaaaacttcatttttaatttaaaaggatctaggtgaagatcctttttgataatctcatgaccaaaatcccttaacgtg
agttttcgttccactgagcgtcagaccccgtagaaaagatcaaaggatcttcttgagatcctttttttctgcgcgtaatctgctgc
ttgcaaacaaaaaaaccaccgctaccagcggtggtttgtttgccggatcaagagctaccaactctttttccgaaggtaactgg
cttcagcagagcgcagataccaaatactgtccttctagtgtagccgtagttaggccaccacttcaagaactctgtagcaccg

cctacatacctcgctctgctaatcctgttaccagtggctgctgccagtggcgataagtcgtgtcttaccgggttggactcaag
acgatagttaccggataaggcgcagcggtcgggctgaacggggggttcgtgcacacagcccagcttggagcgaacgac
ctacaccgaactgagatacctacagcgtgagctatgagaaagcgccacgcttcccgaagggagaaaggcggacaggtat
ccggtaagcggcagggtcggaacaggagagcgcacgagggagcttccagggggaaacgcctggtatctttatagtcctg
tcgggtttcgccacctctgacttgagcgtcgatttttgtgatgctcgtcaggggggcggagcctatggaaaaacgccagcaa
cgcggcctttttacggttcctggccttttgctggccttgaagctgtccctgatggtcgtcatctacctgcctggacagcatggc
ctgcaacgcgggcatcccgatgccgccggaagcgagaagaatcataatggggaaggccatccagcctcgcgtcgaact
agatgatccggctgtggaatgtgtgtcagttagggtgtggaaagtccccaggctccccagcaggcagaagtatgcaaagc
atg

epiCRISPRn sequence (human U6 promoter colored in green, gRNA scaffold colored in blue, BspQI restriction sites underlined, EF1a promoter colored in red, SpCas9 colored in purple, D10A (a-c) mutation colored in yellow, P2A sequences colored in pink, Puromycin resistance gene colored in bright blue, copGFP colored in bright green, EBNA1/ OriP sequence colored in grey.

cattatggtacctttcagacccacctcccaaccccgaggggacccagagagggcctatttcccatgattccttcatatttgcat
atacgatacaaggctgttagagagataattagaattaatttgactgtaaacacaaagatattagtacaaaatacgtgacgtaga
aagtaataatttcttgggtagtttgcagttttaaaattatgttttaaaatggactatcatatgcttaccgtaacttgaaagtatttcga
tttcttggctttatatatcttgtggaaaggacgaaacaccgagaagagcgatgctcttcggttttagagctagaaatagcaagtt
aaaataaggctagtccgttatcaacttgaaaaagtggcaccgagtcggtgctttttttgaattctagatcttgagacaaatggca
gtattcatccacaattttaaaagaaaaggggggattggggggtacagtgcaggggaaagaatagtagacataatagcaac
agacatacaaactaaagaattacaaaaacaaattacaaaaattcaaaattttcgggtttattacagggacagcagagatccac
tttggcgccggctcgagtggctccggtgcccgtcagtgggcagagcgcacatcgcccacagtccccgagaagttgggggg
aggggtcggcaattgaaccggtgcctagagaaggtggcgcggggtaaactgggaaagtgatgtcgtgtactggctccg
cctttttcccgagggtgggggagaaccgtatataagtgcagtagtcgccgtgaacgttctttttcgcaacgggtttgccgcca
gaacacaggtgtcgtgacgcgggatccgccaccatggattacaaagacgatgacgataagatggccccaaagaagaag
cggaaggtcggtatccacggagtcccagcagccgacaagaagtacagcatcggcctggccatcggcaccaactctgtgg
gctgggccgtgatcaccgacgagtacaaggtgcccagcaagaaattcaaggtgctgggcaacaccgaccggcacagca
tcaagaagaacctgatcggagccctgctgttcgacagcggcgaaacagccgaggccacccggctgaagagaaccgcca
gaagaagatacaccagacggaagaaccggatctgctatctgcaagagatcttcagcaacgagatggccaaggtggacga
cagcttcttccacagactggaagagtccttcctggtggaagaggataagaagcacgagcggcaccccatcttcggcaaca
tcgtggacgaggtggcctaccacgagaagtaccccaccatctaccacctgagaaagaaactggtggacagcaccgacaa
ggccgacctgcggctgatctatctggccctggcccacatgatcaagttccggggccacttcctgatcgagggcgacctga
accccgacaacagcgacgtggacaagctgttcatccagctggtgcagacctacaaccagctgttcgaggaaaaccccatc
aacgccagcggcgtggacgccaaggccatcctgtctgccagactgagcaagagcagacggctggaaaatctgatcgcc
cagctgcccggcgagaagaagaatggcctgttcggcaacctgattgccctgagcctgggcctgacccccaacttcaaga
gcaacttcgacctggccgaggatgccaaactgcagctgagcaaggacacctacgacgacgacctggacaacctgctgg
cccagatcggcgaccagtacgccgacctgtttctggccgccaagaacctgtccgacgccatcctgctgagcgacatcctg
agagtgaacaccgagatcaccaaggcccccctgagcgcctctatgatcaagagatacgacgagcaccaccaggacctg
accctgctgaaagctctcgtgcggcagcagctgcctgagaagtacaaagagattttcttcgaccagagcaagaacggcta
cgccggctacattgacggcggagccagccaggaagagttctacaagttcatcaagcccatcctggaaaagatggacggc
accgaggaactgctcgtgaagctgaacagagaggacctgctgcggaagcagcggaccttcgacaacggcagcatcccc
caccagatccacctgggagagctgcacgccattctgcggcggcaggaagatttttacccattcctgaaggacaaccggga

aaagatcgagaagatcctgaccttccgcatcccctactacgtgggccctctggccaggggaaacagcagattcgcctgga
tgaccagaaagagcgaggaaaccatcacccccctggaacttcgaggaagtggtggacaagggcgcttccgcccagagctt
catcgagcggatgaccaacttcgataagaacctgcccaacgagaaggtgctgcccaagcacagcctgctgtacgagtact
tcaccgtgtataacgagctgaccaaagtgaaatacgtgaccgagggaatgagaaagcccgccttcctgagcggcgagca
gaaaaaggccatcgtggacctgctgttcaagaccaaccggaaagtgaccgtgaagcagctgaaagaggactacttcaag
aaaatcgagtgcttcgactccgtggaaatctccggcgtggaagatcggttcaacgcctccctgggcacataccacgatctg
ctgaaaattatcaaggacaaggacttcctggacaatgaggaaaacgaggacattctggaagatatcgtgctgaccctgaca
ctgtttgaggacagagagatgatcgaggaacggctgaaaacctatgcccacctgttcgacgacaaagtgatgaagcagct
gaagcggcggagatacaccggctggggcaggctgagccggaagctgatcaacggcatccgggacaagcagtccggc
aagacaatcctggatttcctgaagtccgacggcttcgccaacagaaacttcatgcagctgatccacgacgacagcctgacc
tttaaagaggacatccagaaagcccaggtgtccggccagggcgatagcctgcacgagcacattgccaatctggccggca
gccccgccattaagaagggcatcctgcagacagtgaaggtggtggacgagctcgtgaaagtgatgggccggcacaagc
ccgagaacatcgtgatcgaaatggccagagagaaccagaccacccagaagggacagaagaacagccgcgagagaat
gaagcggatcgaagagggcatcaaagagctgggcagccagatcctgaaagaacaccccgtggaaaacacccagctgc
agaacgagaagctgtacctgtactacctgcagaatgggcgggatatgtacgtggaccaggaactggacatcaaccggctg
tccgactacgatgtggaccatatcgtgcctcagagctttctgaaggacgactccatcgacaacaaggtgctgaccagaagc
gacaagaaccggggcaagagcgacaacgtgccctccgaagaggtcgtgaagaagatgaagaactactggcggcagct
gctgaacgccaagctgattacccagagaaagttcgacaatctgaccaaggccgagagaggcggcctgagcgaactggat
aaggccggcttcatcaagagacagctggtggaaacccggcagatcacaaagcacgtggcacagatcctggactcccgg
atgaacactaagtacgacgagaatgacaagctgatccgggaagtgaaagtgatcaccctgaagtccaagctggtgtccga
tttccggaaggatttccagttttacaaagtgcgcgagatcaacaactaccaccacgcccacgacgcctacctgaacgccgt
cgtgggaaccgccctgatcaaaaagtaccctaagctggaaagcgagttcgtgtacggcgactacaaggtgtacgacgtgc
ggaagatgatcgccaagagcgagcaggaaatcggcaaggctaccgccaagtacttcttctacagcaacatcatgaacttttt
tcaagaccgagattaccctggccaacggcgagatccggaagcggcctctgatcgagacaaacggcgaaaccggggag
atcgtgtgggataagggccgggattttgccaccgtgcggaaagtgctgagcatgccccaagtgaatatcgtgaaaaagac
cgaggtgcagacaggcggcttcagcaaagagtctatcctgcccaagaggaacagcgataagctgatcgccagaaagaa
ggactgggaccctaagaagtacggcggcttcgacagccccaccgtggcctattctgtgctggtggtggccaaagtggaaa
agggcaagtccaagaaactgaagagtgtgaaagagctgctggggatcaccatcatggaaagaagcagcttcgagaaga
atcccatcgactttctggaagccaagggctacaaagaagtgaaaaaggacctgatcatcaagctgcctaagtactccctgtt
cgagctggaaaacggccggaagagaatgctggcctctgccggcgaactgcagaagggaaacgaactggccctgccctc
caaatatgtgaacttcctgtacctggccagccactatgagaagctgaagggctcccccgaggataatgagcagaaacagct
gtttgtggaacagcacaagcactacctggacgagatcatcgagcagatcagcgagttctccaagagagtgatcctggccg
acgctaatctggacaaagtgctgtccgcctacaacaagcaccgggataagcccatcagagagcaggccgagaatatcatc
cacctgtttaccctgaccaatctgggagcccctgccgccttcaagtactttgacaccaccatcgaccggaagaggtacacc
agcaccaaagaggtgctggacgccaccctgatccaccagagcatcaccggcctgtacgagacacggatcgacctgtctc
agctgggaggcgacaagcgtcctgctgctactaagaaagctggtcaagctaagaaaaagaaagctagcggcagcggcg
ccaccaacttcagcctgctgaagcaggccggcgacgtggaggagaaccccggccccatgaccgagtacaagcccacg
gtgcgcctcgccacccgcgacgacgtccccagggccgtacgcaccctcgccgccgcgttcgccgactaccccgccacg
cgccacaccgtcgatccggaccgccacatcgagcgggtcaccgagctgcaagaactcttcctcacgcgcgtcgggctcg
acatcggcaaggtgtgggtcgcggacgacggcgcggccgtggcggtctggaccacgccggagagcgtcgaagcggg
ggcggtgttcgccgagatcggcccgcgcatggccgagttgagcggttcccggctggccgcgcagcaacagatggaagg
cctcctggcgccgcaccggcccaaggagcccgcgtggttcctggccaccgtcggagtctcgcccgaccaccagggcaa
gggtctgggcagcgccgtcgtgctccccggagtggaggcggccgagcgcgccggggtgcccgccttcctggagacct
ccgcgccccgcaacctccccttctacgagcggctcggcttcaccgtcaccgccgacgtcgaggtgcccgaaggaccgc

gcacctggtgcatgacccgcaagcccggtgccgcgatcgccgaaggatccgcggccgctgagggcagaggaagtcttc
taacatgcggtgacgtggaggagaatcccggcccttccggaatggagagcgacgagagcggcctgcccgccatggaga
tcgagtgccgcatcaccggcaccctgaacggcgtggagttcgagctggtgggcggcggagagggcacccccaagcag
ggccgcatgaccaacaagatgaaaagcaccaaaggcgccctgaccttcagcccctacctgctgagccacgtgatgggct
acggcttctaccacttcggcacctaccccagcggctacgagaaccccttcctgcacgccatcaacaacggcggctacacc
aacacccgcatcgagaagtacgaggacggcggcgtgctgcacgtgagcttcagctaccgctacgaggccggccgcgtg
atcggcgacttcaaggtggtgggcaccggcttccccgaggacagcgtgatcttcaccgacaagatcatccgcagcaacg
ccaccgtggagcacctgcaccccatgggcgataacgtgctggtgggcagcttcgcccgcaccttcagcctgcgcgacgg
cggctactacagcttcgtggtggacagccacatgcacttcaagagcgccatccaccccagcatcctgcagaacgggggc
cccatgttcgccttccgccgcgtggaggagctgcacagcaacaccgagctgggcatcgtggagtaccagcacgccttca
agacccccatcgccttcgccagatcccgcgctcagtcgtccaattctgccgtggacggcaccgccggacccggctccacc
ggatctcgctagagctgaatctaagtcgacttaagaaccgctcgaggccggcaaggccggatccagacatgataagatac
attgatgagtttggacaaaccacaactagaatgcagtgaaaaaaatgctttatttgtgaaatttgtgatgctattgctttatttgta
accattataagctgcaataaacaagttaacaacaacaattgcattcattttatgtttcaggttcagggggaggtgtgggaggttt
tttaaagcaagtaaaacctctacaaatgtggtatggctgattatgatccggctgcctcgcgcgtttcggtgatgacggtgaaa
acctctgacacatgcagctcccggagacggtcacagcttgtctgtaagcggatgccgggagcagacaagcccgtcaggg
cgcgtcagcgggtgttggcgggtgtcggggcgcagccatgaggtcgactctagaggatcgatgccccgccccggacga
actaaacctgactacgacatctctgccccttcttcgcggggcagtgcatgtaatcccttcagttggttggtacaacttgccaac
tgggccctgttccacatgtgacacggggggggaccaaacacaaaggggttctctgactgtagttgacatccttataaatgga
tgtgcacatttgccaacactgagtggctttcatcctggagcagactttgcagtctgtggactgcaacacaacattgcctttatgt
gtaactcttggctgaagctcttacaccaatgctgggggacatgtacctcccaggggcccaggaagactacgggaggctac
accaacgtcaatcagaggggcctgtgtagctaccgataagcggaccctcaagagggcattagcaatagtgtttataaggcc
cccttgttaaccctaaacgggtagcatatgcttcccgggtagtagtatatactatccagactaaccctaattcaatagcatatgt
tacccaacgggaagcatatgctatcgaattagggttagtaaaagggtcctaaggaacagcgatatctcccaccccatgagc
tgtcacggttttatttacatggggtcaggattccacgagggtagtgaaccattttagtcacaagggcagtggctgaagatcaa
ggagcgggcagtgaactctcctgaatcttcgcctgcttcttcattctccttcgtttagctaatagaataactgctgagttgtgaac
agtaaggtgtatgtgaggtgctcgaaaacaaggtttcaggtgacgcccccagaataaaatttggacgggggggttcagtggt
ggcattgtgctatgacaccaatataaccctcacaaaccccttgggcaataaatactagtgtaggaatgaaacattctgaatatc
tttaacaatagaaatccatggggtggggacaagccgtaaagactggatgtccatctcacacgaatttatggctatgggcaac
acataatcctagtgcaatatgatactggggttattaagatgtgtcccaggcagggaccaagacaggtgaaccatgttgttaca
ctctatttgtaacaaggggaaagagagtggacgccgacagcagcggactccactggttgtctctaacacccccgaaaatta
aacggggctccacgccaatggggcccataaacaaagacaagtggccactcttttttttgaaattgtggagtgggggcacgc
gtcagcccccacacgccgccctgcggttttggactgtaaaataagggtgtaataacttggctgattgtaaccccgctaacca
ctgcggtcaaaccacttgcccacaaaaccactaatggcaccccggggaatacctgcataagtaggtgggcgggccaaga
taggggcgcgattgctgcgatctggaggacaaattacacacacttgcgcctgagcgccaagcacagggttgttggtcctca
tattcacgaggtcgctgagagcacggtgggctaatgttgccatgggtagcatatactacccaaatatctggatagcatatgct
atcctaatctatatctgggtagcataggctatcctaatctatatctgggtagcatatgctatcctaatctatatctgggtagtatat
gctatcctaatttatatctgggtagcataggctatcctaatctatatctgggtagcatatgctatcctaatctatatctgggtagtat
atgctatcctaatctgtatccgggtagcatatgctatcctaatagagattagggtagtatatgctatcctaatttatatctgggtag
catatactacccaaatatctggatagcatatgctatcctaatctatatctgggtagcatatgctatcctaatctatatctgggtagc
ataggctatcctaatctatatctgggtagcatatgctatcctaatctatatctgggtagtatatgctatcctaatttatatctgggta
gcataggctatcctaatctatatctgggtagcatatgctatcctaatctatatctgggtagtatatgctatcctaatctgtatccgg
gtagcatatgctatcctcatgcatatacagtcagcatatgatacccagtagtagagtgggagtgctatcctttgcatatgccgc
cacctcccaagggggcgtgaattttcgctgcttgtccttttcctgctggttgctcccattcttaggtgaatttaaggaggccagg

ctaaagccgtcgcatgtctgattgctcaccaggtaaatgtcgctaatgttttccaacgcgagaaggtgttgagcgcggagct
gagtgacgtgacaacatgggtatgcccaattgccccatgttgggaggacgaaaatggtgacaagacagatggccagaaat
acaccaacagcacgcatgatgtctactggggatttattctttagtgcgggggaatacacggcttttaatacgattgagggcgt
ctcctaacaagttacatcactcctgcccttcctcaccctcatctccatcacctccttcatctccgtcatctccgtcatcaccctcc
gcggcagccccttccaccataggtggaaaccagggaggcaaatctactccatcgtcaaagctgcacacagtcaccctgat
attgcaggtaggagcgggctttgtcataacaaggtccttaatcgcatccttcaaaacctcagcaaatatatgagtttgtaaaaa
gaccatgaaataacagacaatggactcccttagcgggccaggttgtgggccgggtccaggggccattccaaaggggaga
cgactcaatggtgtaagacgacattgtggaatagcaagggcagttcctcgccttaggttgtaaagggaggtcttactacctc
catatacgaacacaccggcgacccaagttccttcgtcggtagtcctttctacgtgactcctagccaggagagctcttaaacct
tctgcaatgttctcaaatttcgggttggaacctccttgaccacgatgctttccaaaccaccctcctttttgcgcctgcctccatc
accctgaccccggggtccagtgcttgggccttctcctgggtcatctgcggggccctgctctatcgctcccgggggcacgtc
aggctcaccatctgggccaccttcttggtggtattcaaaataatcggcttcccctacagggtggaaaaatggccttctacctg
gagggggcctgcgcggtggagacccggatgatgatgactgactactgggactcctgggcctcttttctccacgtccacga
cctctccccctggctctttcacgacttccccccctggctctttcacgtcctctaccccggcggcctccactacctcctcgaccc
cggcctccactacctcctcgaccccggcctccactgcctcctcgaccccggcctccacctcctgctcctgcccctcctgctc
ctgcccctcctcctgctcctgcccctcctgcccctcctgctcctgcccctcctgcccctcctgctcctgcccctcctgcccctc
ctgctcctgcccctcctgcccctcctcctgctcctgcccctcctgcccctcctcctgctcctgcccctcctgcccctcctgctc
ctgcccctcctgcccctcctgctcctgcccctcctgcccctcctgctcctgcccctcctgctcctgcccctcctgctcctgccc
ctcctgctcctgcccctcctgcccctcctgcccctcctcctgctcctgcccctcctgctcctgcccctcctgcccctcctgccc
ctcctgctcctgcccctcctcctgctcctgcccctcctgcccctcctgcccctcctcctgctcctgcccctcctgcccctcctcc
tgctcctgcccctcctcctgctcctgcccctcctgcccctcctgcccctcctcctgctcctgcccctcctgcccctcctcctgct
cctgcccctcctcctgctcctgcccctcctgcccctcctgcccctcctcctgctcctgcccctcctcctgctcctgcccctcct
gcccctcctgcccctcctgcccctcctcctgctcctgcccctcctcctgctcctgcccctcctgctcctgcccctcccgctcct
gctcctgctcctgttccaccgtgggtccctttgcagccaatgcaacttggacgtttttggggtctccggacaccatctctatgtc
ttggccctgatcctgagccgcccggggctcctggtcttccgcctcctcgtcctcgtcctcttccccgtcctcgtccatggttat
cacccccctcttctttgaggtccactgccgccggagccttctggtccagatgtgtctcccttctctcctaggccatttccaggtcc
tgtacctggcccctcgtcagacatgattcacactaaaagagatcaatagacatctttattagacgacgctcagtgaatacagg
gagtgcagactcctgccccctccaacagccccccaccctcatccccttcatggtcgctgtcagacagatccaggtctgaa
aattccccatcctccgaaccatcctcgtcctcatcaccaattactcgcagcccggaaaactcccgctgaacatcctcaagatt
tgcgtcctgagcctcaagccaggcctcaaattcctcgtcccccttttttgctggacggtagggatggggattctcgggacccc
tcctcttcctcttcaaggtcaccagacagagatgctactggggcaacggaagaaaagctgggtgcggcctgtgaggatca
gcttatcgatgataagctgtcaaacatgagaattcttgaagacgaaagggcctcgtgatacgcctatttttataggttaatgtca
tgataataatggtttcttagacgtcaggtggcacttttcggggaaatgtgcgcggaacccctatttgtttatttttctaaatacatt
caaatatgtatccgctcatgagacaataaccctgataaatgcttcaataatattgaaaaaggaagagtatgagtattcaacattt
ccgtgtcgcccttattcccttttttgcggcattttgccttcctgtttttgctcacccagaaacgctggtgaaagtaaaagatgctg
aagatcagttgggtgcacgagtgggttacatcgaactggatctcaacagcggtaagatccttgagagttttcgccccgaaga
acgttttccaatgatgagcacttttaaagttctgctatgtggcgcggtattatcccgtgttgacgccgggcaagagcaactcgg
tcgccgcatacactattctcagaatgacttggttgagtactcaccagtcacagaaaagcatcttacggatggcatgacagtaa
gagaattatgcagtgctgccataaccatgagtgataacactgcggccaacttacttctgacaacgatcggaggaccgaagg
agctaaccgcttttttgcacaacatgggggatcatgtaactcgccttgatcgttgggaaccggagctgaatgaagccatacc
aaacgacgagcgtgacaccacgatgcctgcagcaatggcaacaacgttgcgcaaactattaactggcgaactacttactct
agcttcccggcaacaattaatagactggatggaggcggataaagttgcaggaccacttctgcgctcggcccttccggctgg
ctggtttattgctgataaatctggagccggtgagcgtgggtctcgcggtatcattgcagcactggggccagatggtaagccc
tcccgtatcgtagttatctacacgacggggagtcaggcaactatggatgaacgaaatagacagatcgctgagataggtgcc

tcactgattaagcattggtaactgtcagaccaagtttactcatatatactttagattgatttaaaacttcatttttaatttaaaaggat
ctaggtgaagatcctttttgataatctcatgaccaaaatcccttaacgtgagttttcgttccactgagcgtcagaccccgtagaa
aagatcaaaggatcttcttgagatcctttttttctgcgcgtaatctgctgcttgcaaacaaaaaaaccaccgctaccagcggtg
gtttgtttgccggatcaagagctaccaactctttttccgaaggtaactggcttcagcagagcgcagataccaaatactgtcctt
ctagtgtagccgtagttaggccaccacttcaagaactctgtagcaccgcctacatacctcgctctgctaatcctgttaccagtg
gctgctgccagtggcgataagtcgtgtcttaccgggttggactcaagacgatagttaccggataaggcgcagcggtcggg
ctgaacggggggttcgtgcacacagcccagcttggagcgaacgacctacaccgaactgagatacctacagcgtgagcta
tgagaaagcgccacgcttcccgaagggagaaaggcggacaggtatccggtaagcggcagggtcggaacaggagagc
gcacgagggagcttccagggggaaacgcctggtatctttatagtcctgtcgggtttcgccacctctgacttgagcgtcgattt
ttgtgatgctcgtcaggggggcggagcctatggaaaaacgccagcaacgcggcctttttacggttcctggccttttgctggc
cttgaagctgtccctgatggtcgtcatctacctgcctggacagcatggcctgcaacgcgggcatcccgatgccgccggaa
gcgagaagaatcataatggggaaggccatccagcctcgcgtcgaactagatgatccggctgtggaatgtgtgtcagttag
ggtgtggaaagtccccaggctccccagcaggcagaagtatgcaaagcatg

gRNAU6 sequence (tracrRNA colored in blue, human U6 promoter colored in green)

cttccgcttcctcgctcactgactcgctgcgctcggtcgttcggctgcggcgagcggtatcagctcactcaaaggcggtaat
acggttatccacagaatcaggggataacgcaggaaagaacatgtgagcaaaaggccagcaaaagcccaggaaccgtaa
aaaggccgcgttgctggcgtttttccataggctccgcccccctgacgagcatcacaaaaatcgacgctcaagtcagaggtg
gcgaaacccgacaggactataaagataccaggcgtttccccctggaagctccctcgtgcgctctcctgttccgaccctgcc
gcttaccggatacctgtccgcctttctcccttcgggaagcgtggcgctttctcatagctcacgctgtaggtatctcagttcggt
gtaggtcgttcgctccaagctgggctgtgtgcacgaaccccccgttcagcccgaccgctgcgccttatccggtaactatcgt
cttgagtccaacccggtaagacacgacttatcgccactggcagcagccactggtaacaggattagcagagcgaggtatgt
aggcggtgctacagagttcttgaagtggtggcctaactacggctacactagaaggacagtatttggtatctgcgctctgctga
agccagttaccttcggaaaaagagttggtagctcttgatccggcaaacaaaccaccgctggtagcggtggtttttttgtttgca
agcagcagattacgcgcagaaaaaaaggatctcaagaagatcctttgatcttttctacggggtctgacgctcagtggaacga
aaactcacgttaagggattttggtcatgagattatcaaaaaggatcttcacctagatccttttaaattaaaaatgaagttttagca
cgtgtcagtcctgctcctcggccacgaagtgcacgcagttgccggccgggtcgcgcagggcgaactcccgcccccacg
gctgctcgccgatctcggtcatggccggcccggaggcgtcccggaagttcgtggacacgacctccgaccactcggcgta
cagctcgtccaggccgcgcacccacacccaggccagggtgttgtccggcaccacctggtcctggaccgcgctgatgaac
agggtcacgtcgtcccggaccacaccggcgaagtcgtcctccacgaagtcccgggagaacccgagccggtcggtccag
aactcgaccgctccggcgacgtcgcgcgcggtgagcaccggaacggcactggtcaacttggccatggtggccctcctca
cgtgctattattgaagcatttatcagggttattgtctcatgagcggatacatatttgaatgtatttagaaaaataaacaaatagggg
gttccgcgcacatttccccgaaaagtgccacctgtatgcggtgtgaaataccgcacagatgcgtaaggagaaaataccgca
tcaggaaattgtaagcgttaataattcagaagaactcgtcaagaaggcgatagaaggcgatgcgctgcgaatcgggagcg
gcgataccgtaaagcacgaggaagcggtcagcccattcgccgccaagctcttcagcaatatcacgggtagccaacgctat
gtcctgatagcggtccgccacacccagccggccacagtcgatgaatccagaaaagcggccattttccaccatgatattcgg
caagcaggcatcgccatgggtcacgacgagatcctcgccgtcgggcatgctcgccttgagcctggcgaacagttcggct
ggcgcgagcccctgatgctcttcgtccagatcatcctgatcgacaagaccggcttccatccgagtacgtgctcgctcgatgc
gatgtttcgcttggtggtcgaatgggcaggtagccggatcaagcgtatgcagccgccgcattgcatcagccatgatggata
ctttctcggcaggagcaaggtgagatgacaggagatcctgccccggcacttcgcccaatagcagccagtcccttcccgctt
cagtgacaacgtcgagcacagctgcgcaaggaacgcccgtcgtggccagccacgatagccgcgctgcctcgtcttgcag
ttcattcagggcaccggacaggtcggtcttgacaaaaagaaccgggcgcccctgcgctgacagccggaacacggcggc
atcagagcagccgattgtctgttgtgcccagtcatagccgaatagcctctccacccaagcggccggagaacctgcgtgca

atccatcttgttcaatcatgcgaaacgatcctcatcctgtctcttgatcagagcttgatcccctgcgccatcagatccttggcgg
cgagaaagccatccagtttactttgcagggcttcccaaccttaccagagggcgccccagctggcaattccggttcgcttgct
gtccataaaaccgcccagtctagctatcgccatgtaagcccactgcaagctacctgctttctctttgcgcttgcgttttcccttgt
ccagatagcccagtagctgacattcatccggggtcagcaccgtttctgcggactggctttctacgtgaaaaggatctaggtg
aagatcctttttgataatctcatgcctgacatttatattccccagaacatcaggttaatggcgtttttgatgtcattttcgcggtggc
tgagatcagccacttcttccccgataacggagaccggcacactggccatatcggtggtcatcatgcgccagctttcatcccc
gatatgcaccaccgggtaaagttcacgggagactttatctgacagcagacgtgcactggccaggggggatcaccatccgtc
gccccggcgtgtcaataatatcactctgtacatccacaaacagacgataacggctctctcttttataggtgtaaaccttaaact
gccgtacgtataggctgcgcaactgttgggaagggcgatcggtgcgggcctcttcgctattacgccagctggcgaaaggg
ggatgtgctgcaaggcgattaagttgggtaacgccagggttttcccagtcacgacgttgtaaaacgacggccagtgaattgt
aatacgactcactatagggcgaattgggccctctagatgcatgctcgagctcttcgttttagagctagaaatagcaagttaaa
ataaggctagtccgttatcaacttgaaaaagtggcaccgagtcggtgctttttttgtcgactggatccggtaccaaggtcggg
caggaagagggcctatttcccatgattccttcatatttgcatatacgatacaaggctgttagagagataattagaattaatttga
ctgtaaacacaaagatattagtacaaaatacgtgacgtagaaagtaataatttcttgggtagtttgcagttttaaaattatgtttta
aaatggactatcatatgcttaccgtaacttgaaagtatttcgatttcttggctttatatatcttgtggaaaggacgaaacaccgtg
agacgtaactcgtctcggttttagagctagaaatagcaagttaaaataaggctagtccgttatcaacttgaaaaagtggcacc
gagtcggtgctttttttctagacccagctttcttgtacaaagttggcattaaagggcgaattccagcacactggcggccgttact
agtggatccgagctcggtaccaagcttgatgcatagcttgagtattctatagtgtcacctaaatagcttggcgtaatcatggtc
atagctgtttcctgtgtgaaattgttatccgctcacaattccacacaacatacgagccggaagcataaagtgtaaagcctggg
gtgcctaatgagtgagctaactcacattaattgcgttgcgctcactgcccgctttccagtcgggaaacctgtcgtgccagctg
cattaatgaatcggccaacgcgcggggagaggcggtttgcgtattgggcgct

Table S1. Summary of indel Rates for the RFLP Assay

		Day 5	Day 10	Day 15
Cell line	Gene name	Indel %	Indel %	Indel %
H9	*DYRK1A* (SK)	37±3.2	100.0±0.0	100.0±0.0
iPSC1	*DYRK1A* (SK)	32.4±6.4	87.3±5.3	93.8±1.9
iPSC2	*DYRK1A* (SK)	30.5±4.8	86.1±3.4	90.9±1.8
H9	*AAVS1* (SK)	18.6±2.3	83.1±3.8	92.6±2.5
iPSC1	*AAVS1* (SK)	18.9±2.5	74.3±6.3	86.4±4.3
iPSC2	*AAVS1* (SK)	18±1. 7	79.3±4.2	87±5.6
H9	*EMX1* (SK)	13.5± 3.7	69.5±7.8	80.0±5.0
iPSC1	*EMX1* (SK)	12.3±2.3	45.5±4.3	66.0±1.7
iPSC2	*EMX1* (SK)	10±2.5	58.0±3.2	63.0±5.3

H9	*VEGFA* (SK)	31.5±3.9	76.7±5.7	82±2.6
iPSC1	*VEGFA* (SK)	17.8±3.6	48.1±6.5	68.6±2.8
iPSC2	*VEGFA* (SK)	20.7±2.7	66.0±3.6	74.2±4.0
H9	*APC1* (SK)	14.8±6.3	52.5±3.2	63.0±4.8
iPSC1	*APC1* (SK)	12.6±2.5	43.8±2.7	58.6±3.2
iPSC2	*APC1* (SK)	11.4±2.0	43.8±2.6	60.7±4.7
H9	*MLH1* (SK)	17.5±1.3	40.0±2.0	47.0±3.7
iPSC1	*MLH1* (SK)	8.6±2.0	26.0±3.0	38.0±5.9
iPSC2	*MLH1* (SK)	13.4±3.2	31.6±2.3	41.1±6.7
H9	*EMX1* (DK)	12.5±2.4	54.6±5.3	60.8±3.9
iPSC1	*EMX1* (DK)	11.5±3.3	42.6±4.1	53.3±2.9
iPSC2	*EMX1* (DK)	12.4±1.1	45.0±1.5	58.3±3.5
H9	*DYRK1A* (DK)	25.3±4.7	89.8±2.6	91.7±2.0
iPSC1	*DYRK1A* (DK)	24.7±4.9	81.7±2.9	87.8±2.5
iPSC2	*DYRK1A* (DK)	26.1±1.4	83.4±1.2	89.8±1.7
H9	*AAVS1* (DK)	20.5±3.0	73.8±6.4	85.4±4.2
iPSC1	*AAVS1* (DK)	18.5±1.9	67.4±5.6	80.5±4.4
iPSC2	*AAVS1* (DK)	19.0±2.7	68.7±6.2	83.2±4.3
H9	*VEGFA* (DK)	12.8±3.2	47.4±8. 0	60.3±5. 8
iPSC1	*VEGFA* (DK)	14.2±4. 7	42.4±3. 2	46.6±6. 4
iPSC2	*VEGFA* (DK)	13.6±3. 4	44.4±3. 2	49.6±3.0
H9	*APC1* (DK)	12.5±3.2	40.3±2.8	50.1±2.6
iPSC1	*APC1* (DK)	13.0±3. 5	35.3±1.4	41.5± 1.2
iPSC2	*APC1* (DK)	14.5±2. 5	37.8±1.7	45.9±1.9

H9	*MLH1* (DK)	14.9±1. 8	34.9±1. 8	45.7±4. 3
iPSC1	*MLH1* (DK)	15.4±4. 7	33.5±3. 5	39.4±4. 1
iPSC2	*MLH1* (DK)	12.9±1. 4	30.8±5.1	39.8±3. 4
H9	*EMX1* (DN)	13.5±0. 7	64.9±4. 5	74.9±6. 5
iPSC1	*EMX1* (DN)	16.5±2. 1	53.3±3. 2	60.8±3. 4
iPSC2	*EMX1* (DN)	15.3±4. 6	53.2±6. 0	64.4±3. 0
H9	*DYRK1A* (DN)	15.7±1.5	53.3±5. 5	58.3±4.1
iPSC1	*DYRK1A* (DN)	11.3±5. 6	40.6±3. 7	52.9±6. 2
iPSC2	*DYRK1A* (DN)	13.6±6. 5	45.6±5. 6	56.5±5. 3
H9	*APC1* (DN)	16.4±1.9	48.4±2.3	52.8± 2.2
iPSC1	*APC1* (DN)	10.2±1. 3	37.3± 2.4	45.9±2. 6
iPSC2	*APC1* (DN)	12±1.8	39.1±2. 1	46.3±3.0
H9	*MLH1* (DN)	9.1±2. 0	30.8±3. 4	38.9±4. 6
iPSC1	*MLH1* (DN)	10.1± 2.1	26.2±3. 4	36.7± 5.3
iPSC2	*MLH1* (DN)	9.6±1. 6	29.4±4. 9	36.9±3. 4

SK, single-gene knockout

DK, double-gene knockout

DN, double-nicking

Table S2. List of gRNAs for the genome editing

**Single-gene knockout**	
EMX1	AGGGCTCCCATCACATCAACCGG
AAVS1	AAGAAGACTAGCTGAGCTCTCGG
DYRK1A	CCATCTGAAGGCCAGCAGCATGG
VEGFA	CCGTCTGCACACCCCGGCTCTGG

APC1	TGGCAGGTGAGTGAGGCTGCAGG
MLH1	GGAAACGTCTAGATGCTCAACGG
**Double-gene knockout**	
EMX1<br>*DYRK1A*	AGGGCTCCCATCACATCAACCGG<br>CCATCTGAAGGCCAGCAGCATGG
AAVS1<br>*VEGFA*	AAGAAGACTAGCTGAGCTCTCGG<br>CCGTCTGCACACCCCGGCTCTGG
APC1<br>*MLH1*	TGGCAGGTGAGTGAGGCTGCAGG<br>GGAAACGTCTAGATGCTCAACGG
**Double-nicking**	
DYRK1A	CTCCTACAAGAAGATAAGTGAGG<br>CATGCAAACCTTCATCTGTTCGG
EMX1	TGCGCCACCGGTTGATGTGATGG<br>CACGAAGCAGGCCAATGGGGAGG
APC1	TGGCAGGTGAGTGAGGCTGCAGG<br>CCAGAAGTACGAGCGCCGCCCGG
MLH1	GGAAACGTCTAGATGCTCAACGG<br>CAAAATGTCGTTCGTGGCAGGGG
**Genomic deletion**	
EMX1	AGGCCCCAGTGGCTGCTCTGGGG<br>GGCAGAGTGCTGCTTGCTGCTGG
DYRK1A	GTTCCTTAAATAAGAACTTTAGG<br>GGAGTATCAGAAATGACTATTGG
VEGFA1	GGAGGGCTCACGCCGCGCTCCGG

	GCGGGAGGAAGTCTAGAGCAAGG
PPP1R12C	CGCCCGGCGTGCTGACGTCACGG<br>TTCCTTCCTGGTCCCGTCTCTGG

Table S3. Primers used in this study

Primer name	Target	Primer sequences (5'to3')
Amp-F	eipCRISPR vector	CTGCAACTTTATCCGCCTCC
Amp-R	eipCRISPR vector	TCTGACAACGATCGGAGGAC
PPP1R12C-F1	*PPP1R12C* (for deletion)	TTCATCCCCTTTACCCGGTG
PPP1R12C-R1	*PPP1R12C* (for deletion)	CCCTCCCTATTGCACATGGA
PPP1R12C-F2	*PPP1R12C*<br>(for non-deletion)	CCCCTTACCTCTCTAGTCTGTGC
PPP1R12C-R2	*PPP1R12C*<br>(for non-deletion)	GGCTCCATCGTAAGCAAACC
VEGFA-F1	*VEGFA* (for deletion)	GTCGAGGAAGAGAGAGACGG
VEGFA-R1	*VEGFA* (for deletion)	CCAGACCCAAGACAAATGCC
VEGFA-F2	*VEGFA* (for non-deletion)	CAGGAGGGGACAGATGGATG
VEGFA-R2	*VEGFA* (for non-deletion)	GGCTTCACCCTGACTCCTAG
EMX1-F	*EMX1* (for non-deletion)	CCATCCCCTTCTGTGAATGT
EMX1-R	*EMX1* (for non-deletion)	GGAGATTGGAGACACGGAGA
DYRK1A-F	*DYRK1A* (for non-deletion)	GGAGCTGGTCTGTTGGAGAA
DYRK1A-R	*DYRK1A* (for non-deletion)	TCCCAATCCATAATCCCACGTT
suvDYRK1A-F	*DYRK1A* (indels)	GGAGCTGGTCTGTTGGAGAA
suvDYRK1A-R	*DYRK1A* (indels)	TCCCAATCCATAATCCCACGTT

surAAVS-F	*AAVS1* (indels)	TGCTTTCTTTGCCTGGACAC
surAAVS-R	*AAVS1* (indels)	CTGTCACCAATCCTGTCCCT
suvEMX1-F	*EMX1* (indels)	CCATCCCCTTCTGTGAATGT
suvEMX1-R	*EMX1* (indels)	GGAGATTGGAGACACGGAGA
suvVEGFA-F	*VEGFA* (indels)	CAAAGGACCCCAGTCACTCC
suvVEGFA-R	*VEGFA* (indels)	GAGGAGGGAGCAGGAAAGTG
suvAPC-F	*APC1* (indels)	GGCTGTGGGAAGCCAGCAAC
suvAPC-R	*APC1* (indels)	AAGCCAGGGGCCAACTGGAG
suvMLH-F	*MLH1* (indels)	ATATCCTTCTAGGTAGCGGGCAGTAGCC
suvMLH-R	*MLH1* (indels)	TCTCGGGGGAGAGCGGTAAA
EMXoff1-F	OT-1	TCTGTGCCGTATCTCAATGC
EMXoff1-R	OT-1	TTTCTCAAGAGCTCCTGGGG
EMXoff2-F	OT-2	TGCAAAATCCTCTGCAGACAC
EMXoff2-R	OT-2	GCTCATGGTCTACTGCAAGG
EMXoff3-F	OT-3	AGTCCTAGGGCCTTTTACTGG
EMXoff3-R	OT-3	TAAGTTGTTGAGGGGAGGGG
EMXoff4-F	OT-4	TCATACCTTGGGCCAACTGT
EMXoff4-R	OT-4	AGGCACACTGATGACTTCCT
EMXoff5-F	OT-5	ATGAGCTCGGGACATCACAC
EMXoff5-R	OT-5	TCAACGTCTGGGGATCATGA
EMXoff6-F	OT-6	GTGTATCTGCTGCCCCTGT
EMXoff6-R	OT-6	GTGACCTGCTTCCTGATTGC

SUPPLEMENTAL EXPERIMENTAL PROCEDURES

Embryoid Body (EB) Formation Assay

Human ESCs and iPSCs were collected by collagenase IV treatment (1 µg/ul), resuspended in 20% FBS/DMEM media allowing to form EBs in a six-well plate (Costar 3471) for up to 2 weeks. The EBs were then broken down into smaller clumps using a 200 µl pipet tip and allowed to attach onto gelatin-coated plates for an additional 2 days, followed by fixing and staining for the three embryonic germ layers and trophectoderm.

Analysis of Pluripotency Marker Expression

Human ESC and iPSC colonies plated on 6-well tissue culture plates were fixed in 4% paraformaldehyde at room temperature for 5 min and then permeabilized with 1 mL of 0.5% triton for 10 min. After washing with PBS, cells were incubated with primary antibody (1:100 in PBS) at room temperature for 1 h. The primary antibodies used for pluripotency marker staining were Oct3/4 (Santa Cruz Biotechnology), Sox2 (Biolegend), SSEA-4 (Chemicon), Tra-1–60 (Chemicon), Tra-1–81 (Chemicon), and Nanog (Santa Cruz Biotechnology). After thorough washing with PBS (3 x 5 min), AlexaFluor- conjugated secondary antibodies at a dilution of 1:250 (Santa Cruz Biotechnology) were added for 20 min. To highlight the nuclei, DAPI (1:200) was

added together with secondary antibody. After 3 washes with PBS, immunofluorescent images were taken by fluorescent microscopy.

Teratoma Formation Assay

This assay was performed by Beijing Cellapy (Beijing, China). To detect the pluripotency with *in vivo* teratoma formation assay, one million epiCRISPR-modified iPSCs were suspended in 25 µl PBS, mixed with 25 µl Matrigel, and injected into the subcutaneous regions on the backs of immunodeficient, female SCID mice (n=2 spots per group of mice) (Charles River Laboratories, Wilmington, MA). Sixty days after transplantation, teratomas were explanted, and histological staining was performed to analyze cell differentiation. Teratomas were fixed with 4% paraformaldehyde, set in paraffin, sectioned, and stained with hematoxylin & eosin (H&E). Light microscopy was then used to visualize the sections.
